# Prediction of methylphenidate treatment response for ADHD using conventional and radiomics T1 and DTI features: Secondary analysis of a randomized clinical trial

**DOI:** 10.1016/j.nicl.2024.103707

**Published:** 2024-11-21

**Authors:** Mingshi Chen, Zarah van der Pal, Maarten G. Poirot, Anouk Schrantee, Marco Bottelier, Sandra J.J. Kooij, Henk A. Marquering, Liesbeth Reneman, Matthan W.A. Caan

**Affiliations:** a Department of Radiology and Nuclear Medicine, Amsterdam University Medical Center, Location University of Amsterdam, Amsterdam, the Netherlands; b Department of Biomedical Engineering and Physics, Amsterdam University Medical Center, Location University of Amsterdam, Amsterdam, the Netherlands; c Child Study Center Accare, University Medical Center Groningen, Groningen, the Netherlands; d Department of Psychiatry, Amsterdam University Medical Center, Amsterdam, the Netherlands; e Expertise Center Adult ADHD, PsyQ, The Hague, the Netherlands

**Keywords:** ADHD, MRI, Radiomics, Treatment outcome, Methylphenidate, Machine learning

## Abstract

**Background:**

Attention-Deficit/Hyperactivity Disorder (ADHD) is commonly treated with methylphenidate (MPH). Although highly effective, MPH treatment still has a relatively high non-response rate of around 30%, highlighting the need for a better understanding of treatment response. Radiomics of T1-weighted images and Diffusion Tensor Imaging (DTI) combined with machine learning approaches could offer a novel method for assessing MPH treatment response.

**Purpose:**

To evaluate the accuracy of both conventional and radiomics approaches in predicting treatment response based on baseline T1 and DTI data in stimulant-naive ADHD participants.

**Methods:**

We performed a secondary analysis of a randomized clinical trial (ePOD-MPH), involving 47 stimulant-naive ADHD participants (23 boys aged 11.4 ± 0.4 years, 24 men aged 28.1 ± 4.3 years) who underwent 16 weeks of treatment with MPH. Baseline T1-weighted and DTI MRI scans were acquired. Treatment response was assessed at 8 weeks (during treatment) and one week after cessation of 16-week treatment (post-treatment) using the Clinical Global Impressions − Improvement scale as our primary outcome. We compared prediction accuracy using a conventional model and a radiomics model. The conventional approach included the volume of bilateral caudate, putamen, pallidum, accumbens, and hippocampus, and for DTI the mean fractional anisotropy (FA) of the entire brain white matter, bilateral Anterior Thalamic Radiation (ATR), and the splenium of the corpus callosum, totaling 14 regional features. For the radiomics approach, 380 features (shape-based and first-order statistics) were extracted from these 14 regions. XGBoost models with nested cross-validation were used and constructed for the total cohort (n = 47), as well as children (n = 23) and adults (n = 24) separately. Exact binomial tests were employed to compare model performance.

**Results:**

For the conventional model, balanced accuracy (bAcc) in predicting treatment response during treatment was 63 % for the total cohort, 32 % for children, and 36 % for adults (Area Under the Receiver Operating Characteristic Curve (AUC-ROC): 0.69, 0.33, 0.41 respectively). Radiomics models demonstrated bAcc’s of 68 %, 64 %, and 64 % during treatment (AUC-ROCs of 0.73, 0.62, 0.69 respectively). These predictions were better than chance for both conventional and radiomics models in the total cohort (*p* = 0.04, *p* = 0.003 respectively). The radiomics models outperformed the conventional models during treatment in children only (*p* = 0.02). At post-treatment, performance was markedly reduced.

**Conclusion:**

While conventional and radiomics models performed equally well in predicting clinical improvement across children and adults during treatment, radiomics features offered enhanced structural information beyond conventional region-based volume and FA averages in children. Prediction of symptom improvement one week after treatment cessation was poor, potentially due to the transient effects of stimulant treatment on symptom improvement.

## Highlights

• We used conventional and radiomics MRI models to predict MPH treatment response.
• Both conventional and radiomics models outperformed chance in predicting response during treatment.
• Prediction accuracy dropped significantly post-treatment after one-week washout.
• Baseline basal ganglia radiomics were key predictive features for MPH response.

## 1 Introduction

Attention-Deficit/Hyperactivity Disorder (ADHD) is a neurodevelopmental condition that impacts individuals of all ages, marked by difficulties in maintaining concentration, hyperactivity, and impulsiveness (Boesen et al., 2022). Methylphenidate (MPH), a stimulant medication, is commonly prescribed as a first-line treatment for ADHD (Cortese et al., 2018, Dinis-Oliveira, 2017). MPH is thought to function by blocking dopamine and norepinephrine transporters, which increases the availability of these neurotransmitters in the synaptic cleft (Faraone, 2018), thereby enhancing executive function and attention in individuals with ADHD (Mechler et al., 2022). However, MPH shows variable effectiveness, benefiting approximately 70 % of both children and adults diagnosed with ADHD (Cortese et al., 2018, Greenhill et al., 2001, Kooij et al., 2010). Thus, approximately 30 % of individuals with ADHD do not respond to medication, and if responding, only for a limited period of time (Buitelaar and Medori, 2010). Yet, treatment often extends for several years although there are documented risks associated with long-term treatment (Matthijssen et al., 2019). It has been advocated that treating physicians should implement periodic medication-free periods, also in adults with ADHD, to evaluate the benefits of the medication as well as the continuing necessity to distinguish rebound effects and to decide the appropriate duration (van de Loo-Neus et al., 2011). To overcome this trial-and-error approach, individualized treatment strategies have been advocated (National Guideline Centre (UK), 2018, van de Loo-Neus et al., 2011).

Thus, there is a pressing demand to better understand MPH treatment response. MRI techniques, such as T1-weighted imaging and Diffusion Tensor Imaging (DTI), enable the mapping of gray and white matter structures, white matter integrity and structural connectivity in the central nervous system (Lei et al., 2014, Sudre et al., 2023), and can be used to examine MPH treatment effects in individuals with ADHD. Indeed, in our review paper on psychoradiological biomarkers for psychopharmaceutical effects, we noted that diminished volume of the caudate, cerebellum, corpus callosum, and prefrontal cortex all correlate with poorer responses to MPH treatment in children with ADHD (Schrantee et al., 2020). However, the difficulties in defining universal predictors for MPH treatment response arise from several factors. These include the potential insufficiency of subtle volumetric biomarker changes for robust clinical application (Seidman et al., 2011), and the variable brain alterations caused by MPH and its treatment across different age groups, complicating the uniform interpretation of MRI results.

The emergence of artificial intelligence in medical imaging offers new possibilities for identifying common predictors of MPH treatment response. For instance, sophisticated machine-learning approaches could refine the detection of nuanced neuroanatomical and structural connectivity variations in ADHD and its treatment, potentially enhancing the predictive utility of these markers for MPH treatment outcomes (Chang et al., 2021, Kim et al., 2015). Additionally, radiomics is a quantitative approach to medical imaging, which aims to enhance the existing data available to clinicians by means of advanced mathematical analysis. The extracted features, known as radiomics features, can capture various characteristics of tissues or lesions, such as their shape, texture, and intensity (Lambin et al., 2017, Rizzo et al., 2018, van Timmeren et al., 2020). Standard machine learning classification and feature selection techniques, however, tend to display inferior performance in terms of the stability of predictive performance due to the heavy multicollinearity present in radiomics data (Peeters et al., 2019). As one of the machine learning methods, eXtreme Gradient Boosting (XGBoost) uses an iterative approach to reduce errors through decision trees, making it ideal for classifying complex radiomics datasets (Wagner et al., 2021).

Therefore, our study aimed to evaluate the efficacy of both conventional and radiomics approaches in predicting treatment response in treatment-naive individuals with ADHD, using T1-weighted and DTI MRI data. We employed conventional analyses with predefined regions of interest (ROI) from the literature, as well as radiomics analyses with predefined radiomics features. These predictions were evaluated during treatment and one week after treatment cessation.

## 2 Materials and methods

### 2.1 Study design and participants

This study is part of the Effects of Psychotropic Drugs on Developing Brain–Methylphenidate randomized placebo-controlled trial (ePOD-MPH RCT, NTR3103), conducted between October 13, 2011 and June 15, 2015. The original cohort consisted of 50 boys (10–12 years of age) and 49 men (23–40 years of age) diagnosed with ADHD (all subtypes) according to the Diagnostic and Statistical Manual of Mental Disorders, 4th Edition (DSM-IV) (American Psychiatric Association and American Psychiatric Association. Task Force on DSM-IV, 1994). All participants were stimulant treatment-naive at baseline and were eligible for pharmacological treatment with MPH. Participants were assigned to 16 weeks of treatment with MPH or placebo, prescribed by the treating physician under double-blind clinical guidance (reduction of ADHD symptoms) following Dutch treatment guidelines (Schrantee et al., 2016). The study protocol was approved by the Central Committee on Research Involving Human Subjects in The Hague on March 24, 2011 (identifier NL34509.000.10) and subsequently at The Netherlands National Trial Register (identifier NTR3103). Informed written consent was secured from all participants or their guardians. Further details on recruitment, inclusion/exclusion criteria and a Consolidated Standards of Reporting Trials (CONSORT) flow diagram are further shown in I.1 Supplement Methods and Fig. A1.

**Fig. 1:**
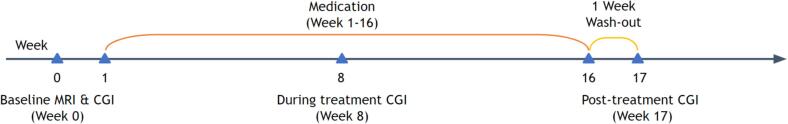
Overview of the study timeline. We utilized MRI measurements taken at baseline, before randomization to treatment. Clinical assessments of ADHD symptom severity, specifically the Clinical Global Impressions (CGI), were conducted at baseline, at 8 weeks (during treatment), and 1 week after the trial concluded (post-treatment).

The primary objective of the ePOD-MPH RCT was to investigate the age-dependent effects of MPH on the outgrowth of the dopaminergic system (Schrantee et al., 2016). The current study reports on secondary analyses, aimed at investigating the value of baseline structural radiomics MRI features associated with good and poor responders to MPH, and therefore solely focused on individuals that were randomized to the MPH treatment condition, consisting of 23 children and 24 adults. The overview of the study timeline is shown in Fig. 1.

### 2.2 Clinical assessment and treatment response

All ADHD participants underwent blinded evaluation of treatment response at week 8 (during treatment) and week 17 (post-treatment) using the Clinical Global Impressions − Improvement (CGI-I) and Severity (CGI-S) scales. These scales evaluate global symptom severity and improvement in both clinical and research settings (Busner and Targum, 2007). For both CGI-I and CGI-S scales, missing scores were imputed using nearest-neighbor interpolation (Schrantee et al., 2016). The CGI-I was used as a measure of treatment response and was rated on a 7-point Likert scale (1=“very much improved”, 2=“much improved”, 3=“minimally improved”, 4=“no change”, 5=“minimally worse”, 6=“much worse”, 7=“very much worse”). Participants were categorized as good treatment responders (CGI-I scores of 1 or 2) or poor treatment responders (CGI-I scores of 3–7). The response rate was calculated as (1.1):

(1.1) Responserate=goodresponders/totalparticipants

As a secondary outcome, we used the CGI-S scale to evaluate global symptom severity during treatment and at post-treatment assessment. The CGI-S is also rated on a 7-point Likert scale (1=“normal, not at all ill”, 2=“borderline mentally ill”, 3=“mildly ill”, 4=“moderately ill”, 5=“markedly ill”, 6=“severely ill”, 7=“among the most extremely ill patients”). Participants were categorized as having low symptom severity (CGI-S scores of 1–––3) or high symptom severity (CGI-S scores of 4–––7). The proportion of low symptom severity rate was calculated as (1.2):

(1.2) Proportionoflowsymptomseverityrate=lowsymptomseverityparticipants/totalparticipants

Our use of the CGI scales ensured consistent assessment across both children and adults, despite the different ADHD-specific symptom scales used for each group. However, we recognize that the CGI scales assess general clinical improvement and symptom severity, rather than specific ADHD symptoms. This presents a limitation, as ADHD-specific scales may offer greater sensitivity to changes in ADHD symptomatology (Woods et al., 2014).

### 2.3 Image Acquisition and Preprocessing

All MRI scans at baseline were acquired using a 3.0 T MRI Achieva or Intera scanners, equipped with an eight-channel sensitivity encoding head coil and body coil transmission, supplied by Philips Medical Systems (Best, Netherlands). First, an anatomical 3D–fast field echo T1-weighted scan was obtained with the following scan parameters: repetition time (TR)/echo time (TE): 9.8/4.6 ms; field of view (FOV): 256 × 256 × 120 mm; voxel size: 0.875 × 0.875 × 1.2 mm. DTI scans were acquired using the following parameters: field of view: TR/TE: 8135/94 ms, FOV: 224 × 224 x 120 mm, voxel size 2 mm^3^, b = 1000 s/mm^2^, 4 non-diffusion weighted images (b = 0 s/mm^2^), 46 gradient directions.

Image quality was determined by calculating the coefficient of joint variation (CJV) and the DTI motion score, with lower scores indicating better image quality (I.2 Supplement Methods). Using T1-weighted scans, gray matter volume was segmented using FastSurfer. ROIs were selected a priori focusing on regions with the strongest evidence related to ADHD pathophysiology and response to MPH, as reviewed by us (Schrantee et al, 2020), i.e., the volume of caudate, putamen, pallidum, and accumbens. Indeed, reduced basal ganglia volume is the most prominent and replicable structural abnormality in ADHD (Nakao et al., 2011). We added the hippocampal volume based on our preclinical work (van der Marel et al., 2015). We separately assessed the left and right hemispheres, totaling 10 ROIs. From the DTI scans, FA values were calculated using Tract-Based Spatial Statistics (TBSS) (Smith et al., 2004) software. We added the same 4 DTI ROIs that we used in our prior work in this cohort on the effects of MPH on FA (Bouziane et al., 2019, van der Marel et al., 2014), including the FA values of the entire brain white matter, bilateral Anterior Thalamic Radiation (ATR), and the splenium of the corpus callosum. For more detailed information on processing steps see Supplement Methods I.3. For the radiomics features, we drew on our prior research (Poirot et al., 2022, Poirot et al., 2024), which demonstrated that shape-based and first-order statistics generally exhibit better reproducibility than higher-order features, limiting our study to only shape-based and first-order statistics features. For DTI data, we included only first-order statistics, as shape-based features are not applicable to skeletonized FA maps in the TBSS analysis.

We conducted three types of analysis: 1) conventional ROI-based t-tests between good and poor responders (14 features, volume of T1 ROIs and the mean FA for DTI), 2) conventional machine learning analysis (14 features, volume of T1 ROIs and the mean FA for DTI), 3) radiomics analysis (380 features, shape-based and first-order statistics in the same 14 ROIs). Additional details are available in I.3 Supplement Methods and Supplement Table A1.

### 2.4 Conventional statistical analysis

Participant characteristics were evaluated using two-sided statistical tests: Pearson Chi-Square and Fisher’s Exact Test for categorical variables for the entire MPH group, children and adult MPH subgroups, respectively, and Mann-Whitney U-tests for continuous variables. Relationships between features and age, features and motion scores, and CJV and motion score were assessed using Spearman Rank Correlation Tests.

A conventional analysis of T1 and DTI feature distributions was performed using Bayesian analysis using Cohen’s d as effect size and 95 % Highest Density Interval (HDI) as confidence interval. Here, the implementation of Bayesian Estimation Supersedes the *t*-test (BEST) was used (Kruschke, 2013, Kruschke and Liddell, 2018). Conventional and radiomics machine learning model performance was evaluated using balanced accuracy (bAcc), precision, recall, F1 score, Receiver Operating Characteristic (ROC) curve, Area Under the ROC Curve (AUC-ROC) value, and AUC of Precision-Recall Curve (AUC-PRC). To understand how the features contribute to the model during training, SHapley Additive exPlanations (SHAP) values were generated to analyze feature contributions and importance (Lundberg et al., 2020). Exact binomial tests were applied to compare the model performances across participant and age-based subgroups and chance (Sundjaja et al., 2023). Statistical significance was set at *p* < 0.05, with analyses conducted using SPSS (v28.0) and Python 3.10. Further details are in I.4 Supplement Methods.

### 2.5 Classification model

**Fig. 2:**
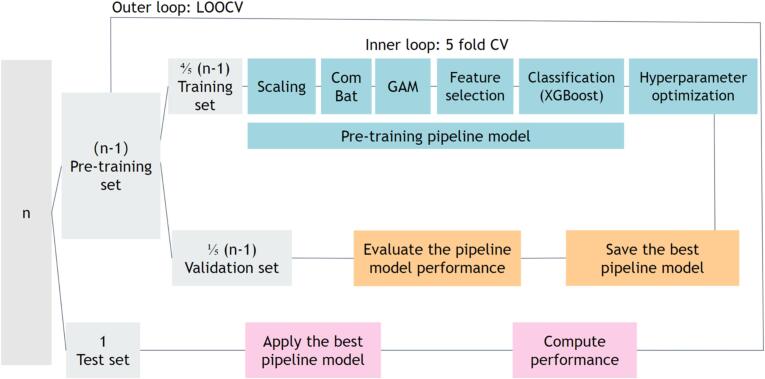
Flowchart of the nested leave-one-out cross-validation (LOOCV) over *n* subjects.

A classification model was built and used for both the conventional analysis of 14 features and the radiomics analysis of 380 features, and detailed in Fig. 2. The classifier was tasked to classify poor and good responders, encoded in a binary outcome variable. The model utilized nested cross-validation with an outer loop of leave-one-out-cross-validation (LOOCV) and an inner loop of 5-fold cross-validation for unbiased performance assessment. Note that by performing scaling, motion regression, feature selection and hyperparameter optimization within a nested cross-validation, we aimed to design a framework robust to overtraining.

Within the inner cross-validation loop, the data was scaled using RobustScaler from sklearn 1.2.2, which adjusts according to the interquartile range (25th to 75th quantile). The DTI motion score and age group (children vs. adults) were incorporated as covariates, considering their potential impact on treatment outcomes. To eliminate the effect of covariates, NeuroComBat-sklearn 0.1.3 (ComBat) was employed to harmonize age difference (Fortin et al., 2018) and Linear Generalised Additive Models (GAM) from pyGAM 0.9.0 for motion score (Servén et al., 2018). ComBat is commonly adopted to correct for site-effects in multicenter studies. Recognizing that age can significantly influence radiomics features, we treated our two age groups as separate entities to enable ComBat to mitigate age-related variability in the radiomics features.

To address the challenge of high-dimensional input data combined with a small sample size, an enhanced feature selection process was adopted in the radiomics analysis. Features were excluded for missing values, low variance (median absolute deviation ≤ 0.001), and high correlation (correlation coefficient ≥ 0.8) in the feature selection process. The most significant features were identified through recursive feature elimination from sklearn 1.2.2, with the total number of retained features based on established criteria (1.3) (Hua et al., 2005):

(1.3) $$\mathrm{Number}\;\mathrm{of}\;\mathrm{features}\;\leq \sqrt{N\;\;\mathrm{samples}}$$

XGBoost 1.7.5 was chosen as the algorithm for both recursive feature elimination and binary outcome prediction. Hyperparameter optimization was achieved via Bayesian Optimisation from skopt 0.9.0 over 50 iterations. Probabilistic sampling (StratifiedShuffleSplit from sklearn 1.2.2) was applied based on balancing positive and negative outcomes (Pedregosa et al., 2012). Weights were added to the XGBoost classification model (Chen and Guestrin, 2016). AUC-ROC was used for model evaluation.

We analyzed binary outcomes from CGI-I scores for 47 MPH-treated participants (total cohort), split into children (n = 23) and adults (n = 24) subgroups. Due to age-based subgroup division, ComBat was not used for age difference harmonization during subgroup analyses. Given the small sample sizes in both positive and negative subgroups, feature selection using recursive feature elimination could be less reliable, making reliance on XGBoost's built-in feature selection mechanism potentially more effective. In our main analysis, we present results using feature selection for the total cohort and without feature selection for the children and adult subgroups. Additionally, we conducted comparisons between models with and without feature selection.

To check for motion artifacts’ impact, scatter plots of DTI motion scores and CJV were inspected, and model robustness was confirmed through reanalysis after excluding outliers (identified by visual inspection). Additionally, to assess the robustness of our models, recalculations were performed after excluding outliers for both the total cohort group and the children group. We applied the same methodology to our secondary outcome measure CGI-S. Further details can be found in I.4 Supplement Methods.

## 3 Results

### 3.1 Participant characteristics

Forty-seven ADHD stimulant treatment-naive participants (23 boys and 24 adult men) were included in this secondary analysis of the ePOD-MPH RCT. Based on the CGI-I scores, during treatment we identified 17 good responders and 30 poor responders in the total cohort (response rate 36.2 %), 7 good responders and 16 poor responders in the children (response rate 30.4 %) and 10 good responders and 14 poor responders in the adults (response rate 41.7 %). Post-treatment, we identified 18 good responders and 29 poor responders in the total cohort (response rate 38.3 %), 3 good responders and 20 poor responders in the children (response rate 13.0 %) and 15 good responders and 9 poor responders in the adults (response rate 62.5 %), see Supplement Table A2. Table 1 details the demographics and pre-treatment clinical characteristics for both good responders and poor responders during treatment and post-treatment. No significant differences in baseline demographics and ADHD characteristics were found between good responders and poor responders in either age group (as defined either during treatment and post-treatment). Additionally, no significant differences were found between good and poor responders in motion scores (total cohort: p = 0.86, children: p = 0.50, adults: p > 0.99) or CJV (total cohort: p = 0.13, children: p = 0.46, adults: p = 0.26). Overall symptom severity scores evaluated using the CGI-S are shown in Supplement Table A3. The proportion of low symptom severity rate was 59.6 % and 48.9 % in the total cohort, 34.8 % and 26.1 % in children, and 83.3 % and 70.8 % in adults during treatment and post-treatment, respectively.

**Table 1:** Demographics and pretreatment clinical characteristics grouped by CGI-I treatment response.

Parameters	During treatment										Post-treatment								
	All participants		Children		Adults		*P* value^a^				All participants		Children		Adults		*P* value^a^		
	Good Responder (n = 17)	Poor Responder (n = 30)	Good Responder (n = 7)	Poor Responder (n = 16)	Good Responder (n = 10)	Poor Responder (n = 14)	All partici- pants	Children	Adults		Good responder (n = 18)	Poor responder (n = 29)	Good responder (n = 3)	Poor responder (n = 20)	Good responder (n = 15)	Poor responder (n = 9)	All partici- pants	Children	Adults
Age (y)^b^	−	−	11 ± 1	11 ± 1	27 ± 3	29 ± 5	−	0.45	0.15		−	−	11 ± 1	11 ± 1	27 (26, 29)	29 ± 5	−	0.83	0.56
Estimated IQ^c^	105 ± 15 (n = 15)	108 ± 17	102 ± 20	108 ± 23	109 ± 10 (n = 8)	107 ± 8	0.72	0.54	0.71		103 ± 10 (n = 16)	109 ± 19	92 ± 10	108 ± 22	106 ± 9 (n = 13)	111 ± 9	0.36	0.31	0.39
ADHD subtype							0.42	1.00	0.24								0.91	1.00	1.00
− Inattentive	7	16	4	8	3	8	−	−	−		9	14	2	10	7	4	−	−	−
− Hyperactive/ impulsive	0	0	0	0	0	0	−	−	−		0	0	0	0	0	0	−	−	−
− Combined	10	14	3	8	7	6	−	−	−		9	15	1	10	8	5	−	−	−
ADHD symptoms^d^																			
− DBD-RS Inattention	−	−	21 ± 2	22 ± 3	−	−	−	0.69	−		−	−	20 ± 3	22 ± 3	−	−	0.27	0.31	−
− DBD-RS Hyperactivity	−	−	15 ± 5	15 ± 5	−	−	−	0.66	−		−	−	15 ± 3	15 ± 6	−	−	0.93	0.97	−
− ADHD-SR	−	−	−		35 ± 8 (n = 8)	33 ± 11 (n = 13)	−	−	0.60		−	−	−		34 ± 10 (n = 13)	33 ± 10 (n = 8)	0.86	−	0.86
Adherence (%)	90 ± 7 (n = 15)	91 (81,97) (n = 28)	89 ± 9 (n = 5)	82 (70,95) (n = 15)	90 ± 6	90 (84, 98) (n = 13)	0.78	0.54	0.61		89 ± 8 (n = 16)	92 (86,97) (n = 27)	82 (75,) (n = 2)	84 (81, 95) (n = 18)	92 (80, 96) (n = 14)	92 ± 9	0.80	0.52	0.40
Response rate (%)^e^	36.2		30.4		41.7		−	−	−		38.3		13.0		62.5		−	−	−

Note: For normally distributed data, characteristics are presented in means ± standard deviations; for non-normally distributed data, characteristics are presented in median (25th percentile, 75th percentile).

^a^*P*-values were obtained with 2-sided, using chi-square tests (Pearson Chi-Square for all MPH groups and Fisher’s Exact Test for children MPH group or adults MPH group) for categorical variables, and. Mann-Whitney U-tests for non-normally distributed continuous variables.

^b^Age is only reported for children and adults separately, because they represent discrete age groups.

^c^IQ = intelligence quotient. For children: Wechsler Intelligence Scale for Children (WISC); for adults: National Adults Reading Test (NART) obtained at baseline; those scores were unavailable for two children and three adults.

^d^For children: DBD-RS = disruptive behavior disorder rating scale; for adults: ADHD-SR = Attention Deficit Hyperactivity Disorder-Self Report.

^e^Response rate = good responders / total participants.

### 3.2 ROI-based analyses of feature distribution

**Fig. 3:**
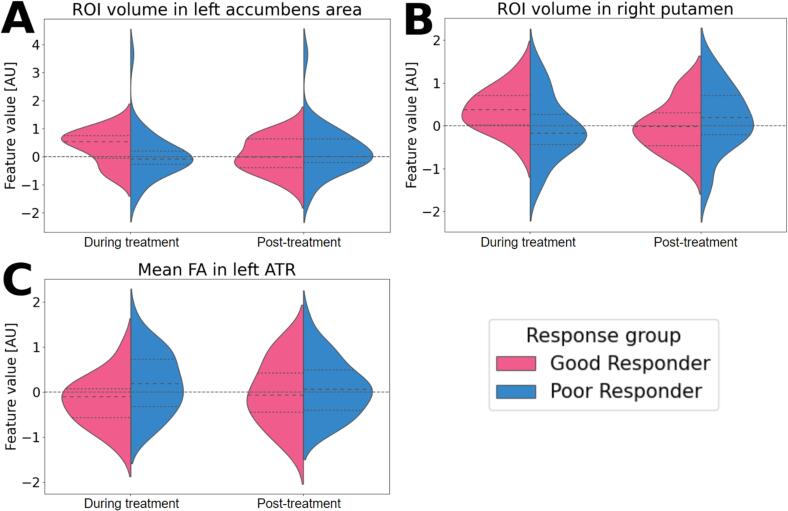
Violin plot of feature values in ROI-based analyses of good and poor responders during treatment and post-treatment with CGI-I (total cohort). Feature values underwent scaling and were harmonized using the NeuroComBat method. A: Left Accumbens regions of interest (ROI) Volume; B: Right Putamen ROI Volume; C: Left Anterior Thalamic Radiation (ATR) Mean Fractional Anisotropy (FA). Dashed line: upper (75th percentile) quartiles, median (50th percentile), and lower (25th percentile) quartiles. AU: arbitrary unit.

ROI-based analyses indicated larger volumes in good compared to poor responders during treatment in the left nucleus accumbens (effect size = 0.60, 95 % HDI: (0.088, 1.20)) and right putamen (effect size = 0.68, 95 % HDI: (0.082, 1.23)) (Fig. 3A, B). Mean FA in the left ATR was lower in good responders compared to poor responders (effect size = -0.58, 95 % HDI: (−1.22, −0.056)) (Fig. 3C). Post-treatment, these effect sizes were lower (left nucleus accumbens volume: −0.10 (−0.70, 0.40); right putamen volume: −0.27 (−0.84, 0.26); left ATR FA: −0.11 (−0.73, 0.46)), but this was not significantly different compared to during treatment. No significant differences were found in other ROI volumes or mean FA values (Supplement Fig. A2, A3).

### 3.3 Conventional model performance on CGI-I

Performance of the conventional model is shown in Table 2. For the total cohort, the conventional model significantly outperformed chance during treatment (bAcc 63 %, AUC-ROC 0.69, *p* = 0.04), but not post-treatment (bAcc 42 %, AUC-ROC 0.34, *p* = 0.77). During treatment, models performed better for the total cohort compared to the children (bAcc 32 %, AUC-ROC 0.33) and adults (bAcc 36 %, AUC-ROC 0.41) separately. The model's performance was significantly worse than chance for children during treatment (*p* = 0.01) but not for adults (*p* = 0.15). Post-treatment, predictive performance significantly declined and did not surpass chance for whole-group and subgroup analyses. As the children subgroup had limited good vs. poor responders (n = 3 vs. n = 20), the conventional model failed to classify.

**Table 2:** Conventional and radiomics model performance with CGI-I as outcome evaluation.

	Conventional model								Conventio-nal model vs. chance		Radiomics model						Radiomics model vs. chance		Conventional vs. Radiomics model
Endpoint	Group		bAcc ^a^ (%)	Precision	Recall	F1 Score	AUC- ROC^b^	AUC- PRC^c^	*P* value^d^		bAcc ^a^ (%)	Precision	Recall	F1 Score	AUC- ROC^b^	AUC-PRC^c^	*P* value^d^		*P* value^d^
During treatment	47 children + adults (FS)^e^		63	0.53	0.53	0.53	0.69	0.55	0.04		68	0.64	0.53	0.58	0.73	0.66	0.003		0.61
	23 children^f^		32	0.21	0.57	0.31	0.33	0.36	0.01		64	0.42	0.71	0.53	0.62	0.39	0.40		0.02
	24 adults^f^		36	0.31	0.50	0.38	0.41	0.48	0.15		64	0.54	0.70	0.61	0.69	0.56	0.31		0.14
Post- treatment	47 children + adults (FS)^e^		42	0.27	0.22	0.24	0.34	0.35	0.77		55	0.42	0.61	0.50	0.56	0.48	0.77		0.68
	23 children^f,g^		−	−	−	−	−	−	−		−	−	−	−	−	−	−		−
	24 adults^f^		49	0.62	0.53	0.57	0.55	0.73	1.00		32	0.50	0.53	0.52	0.21	0.52	0.31		0.51

^a^: bAcc: Balanced accuracy.

^b^: AUC-ROC: Area under the Receiver Operating Characteristic Curve.

^c^: AUC-PRC: Area under the Precision-Recall Curve.

^d^: *P* values were obtained by exact binomial tests.

^e^: FS: feature selection. 7 features were selected in 47 participants (children + adults).

^f^: No ComBat harmonization and no feature selection in subgroup analyses.

^g^: Children subgroup at post-treatment had limited good responders (3/23), and it failed to classify.

SHAP value analysis of the conventional model showed that the most influential features of model prediction across the total cohort included the volume of right putamen and left accumbens area, and mean FA of forceps major and whole brain (Supplement Fig. A4).

### 3.4 Radiomics model performance on CGI-I

**Fig. 4:**
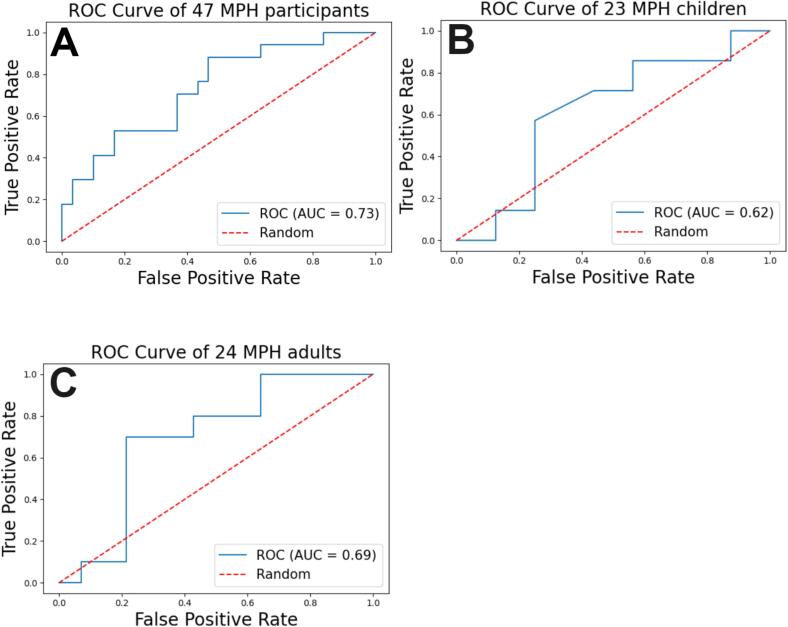
Radiomics models’ ROC curves with MPH group during treatment for CGI-I (A: total cohort; B: children subgroup; C: adult subgroup).

Based on established criteria (dependent on sample size) (Hua et al., 2005), seven features were retained by recursive feature elimination for the total cohort. The performance metrics of the radiomics models are reported in Table 2. During treatment, the radiomics model performed better than chance (bAcc of 68 %, AUC-ROC of 0.73, *p* = 0.003) for the total cohort. Performance slightly surpassed the conventional model, but this was not statistically significant (*p* = 0.61). For the children subgroup, the radiomics model performed significantly better (bAcc of 64 %, AUC-ROC 0.62, *p* = 0.02) than the conventional model (bAcc 32 %, AUC-ROC 0.33). ROC curves of Radiomics models during treatment are displayed in Fig. 4. Similar to the conventional models, the predictive performance of the radiomics models significantly diminished post-treatment, and it failed to classify the children.

**Fig. 5:**
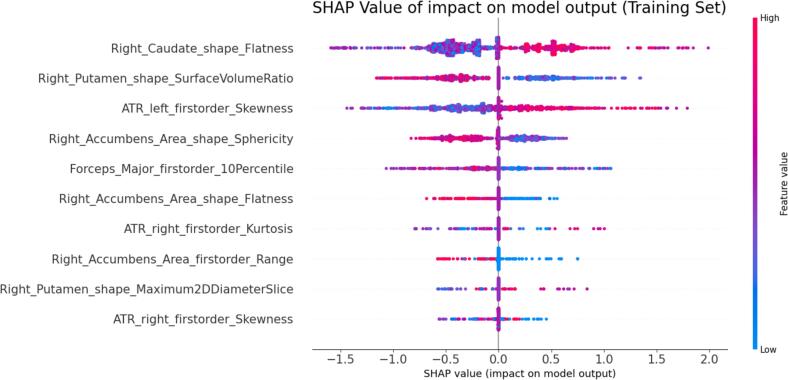
Top 10 SHapley Additive exPlanations (SHAP) values of radiomics models in training set for the total cohort during treatment with CGI-I evaluation. Each dot represents a single data point from the training set, with 46 samples from 47 models generated by LOOCV. Horizontal Axis: SHAP values, which represent the impact of the feature on the model’s output (positive values push the prediction higher, like “1″ for the good responder, while negative values push it lower, like “0″ for the poor responder). Vertical Axis: Features, sorted by their average importance. Color: Indicates the feature value (e.g., blue for low values and red for high values).

SHAP value analysis from the radiomics model also revealed the most impactful features driving predictions throughout the total cohort, including the flatness of right caudate, the surface volume ratio of right putamen, the sphericity of right accumbens area, the skewness of left ATR, and the 10th percentile of forceps major features (Fig. 5). SHAP value analyses for radiomics subgroup analyses are also presented in the Supplement Fig. A5 and A6, respectively.

### 3.5 Sensitivity analyses with conventional and radiomics models and CGI-S

In children, three outliers with high CJV and DTI motion scores were identified (Fig. A7). The Spearman Rank Correlation Test revealed that 380 features from the Radiomics model were highly correlated with age and less so with CJV and DTI motion (Fig. A8). After employing ComBat and GAMs for harmonization, the age correlation was effectively neutralized, though it inadvertently introduced additional correlation with CJV (Fig. A9). The subsequent removal of three outliers successfully eliminated this CJV correlation (Fig. A10).

Sensitivity analyses excluding these outliers revealed a significant impact on both the conventional and radiomics model performance during treatment for the total cohort (conventional model: bAcc 59 %, AUC-ROC 0.59; radiomics model: bAcc 58 %, AUC-ROC 0.57). The performance metrics after excluding these outliers, for both the total cohort and the children subgroup are shown in Supplement Table A4.

Moreover, we compared our models including and excluding an additional feature selection step (see 2.4, Supplement Table A4). Feature selection improved model performance only for the radiomics model in the adult subgroup during treatment, achieving a bAcc of 70 % and an AUC-ROC of 0.89.

Finally, we assessed to what extent our results differ when we use symptom severity (CGI-S) as outcome compared to treatment response (CGI-I) (Supplement Table A5). Contrary to CGI-I, conventional and radiomics models with CGI-S performed better post-treatment compared to during treatment, with the conventional model achieving a bAcc of 66 % and AUC-ROC of 0.63, and the radiomics model reaching a bAcc of 70 % and AUC-ROC of 0.73. At post-treatment, for the adult subgroup, the conventional model with CGI-S demonstrated robust performance, achieving a balanced accuracy (bAcc) of 84 % and an AUC-ROC of 0.77.

## 4 Discussion

This secondary analysis of our RCT with MPH aimed to investigate the value of machine learning approaches using baseline structural brain MRI in predicting clinical improvement in response to MPH treatment in boys and adult men with ADHD. Both our conventional and radiomics predictive models showed significant improvement over chance, but only during treatment, and not one week after cessation of MPH-treatment. The radiomics model only outperformed the conventional model during treatment in the children subgroup.

### 4.1 Analysis of predictive features

In the conventional machine learning model, which included region-based volume and FA averages, we identified that good responders demonstrated larger volumes of the left accumbens and right putamen, along with lower mean FA in the left ATR. In addition, radiomics models identified that the more predictive features were higher right caudate flatness and left ATR skewness, and lower right putamen surface volume ratio and right accumbens area sphericity. These findings suggest that complex morphological characteristics beyond simple volumetric measures may offer additional predictive value in our selected regions of interest, potentially capturing subtle structural variations that contribute to treatment response.

Our finding that significantly larger basal ganglia ROI volumes were associated with good treatment response during treatment, most prominently in the left nucleus accumbens and right putamen, is in line with the literature. Moreno et al. (2014) found that participants who respond positively to MPH treatment tend to have a higher gray matter volume in both caudate nuclei and the right nucleus accumbens, although this study was restricted to pediatric participants only. Also in line with our work, Chang et al. (2021) observed that poor treatment responders (participants with ADHD aged 6–42 years) exhibited smaller volumes of the left putamen and larger volumes in the precuneus at baseline. While our study did not observe hippocampal volume differences between good responders and poor responders, Kim et al. (2022) reported that ADHD youth with poor response to MPH treatment had increased volumes in subregions of the amygdala and hippocampus.

Additionally, in our conventional analysis of DTI data, good responders typically showed lower mean FA in the left ATR. Correspondingly, the radiomics model identified left ATR skewness as a positive predictor of treatment response, indicating a possible connection between left ATR structure at baseline and treatment response. Mazzetti et al. (2022) also noted that before treatment initiation, ADHD symptom severity was associated with lower FA within the ATR. Other work found a correlation between smaller volume in left dorsal superior longitudinal fasciculus in DTI and reduced likelihood of positive response to two-month MPH treatment in adults with ADHD (Parlatini et al., 2023). Additionally, Hung et al. (2024) reported that in better responders, ADHD symptom improvement correlated with pre-treatment structural connectivity strength in the striatum (measured by FA), where weaker connectivity was tied to greater clinical improvement.

### 4.2 During treatment versus post-treatment

Our study demonstrated that the duration of treatment and/or medication status significantly impacts the performance of our CGI-I models: both conventional and radiomics models outperformed chance during treatment, but this was no longer the case at post-treatment assessment. During treatment, participants were titrated to their optimal individual dose, which likely contributed to symptom improvement in cases where MPH was effective. Conversely, at post-treatment, participants underwent a one-week washout period, potentially leading to a sudden worsening of ADHD symptoms. Consequently, the relationship between baseline structural MRI features and treatment response may be more pronounced and detectable during the active treatment phase, when medication effects are at their peak and symptoms are optimally managed (MTA Cooperative Group, 2004).

### 4.3 Response rate

Response rates during treatment evaluated with CGI-I were significantly lower than the typically observed 70 % response rate across age groups (Cortese et al., 2018, Greenhill et al., 2001, Kooij et al., 2010, Retz et al., 2020). One week after treatment cessation, the overall response rate remained similar at 38 % across the total cohort, with a decrease to 13 % in children and an increase to 63 % in adults. Using the CGI-S scale, symptom severity ratings more closely matched those reported in the literature, with overall 60 % during treatment and 49 % at post-treatment assessment (Ginsberg et al., 2014, Ishii-Takahashi et al., 2015).

In contrast to CGI-I, both models with CGI-S evaluation performed better at post-treatment, rather than during treatment, especially in adults. The discrepancy between the prediction models of these two CGI scales is likely associated with their operationalization. For example, an individual that scores 4 on the CGI-S at baseline (“moderately ill”) may experience some symptom improvement, resulting in a 3 (“mildly ill”) during treatment. However, in this situation the individual will likely not score a 1 or 2 on the CGI-I (“very much improved” and “much improved” respectively), and will be therefore categorized as a poor responder. Moreover, we could speculate that ‘low symptom severity’ is marked by different structural brain features than ‘treatment response’, but this remains to be investigated. Finally, it is important to note that the CGI-I scale measures general clinical improvement not specific to ADHD symptomatology (Woods et al., 2014), and therefore ADHD-specific scales like DBD-RS, ADHD-RS, and Conners’ Rating Scales, may offer greater sensitivity. Nonetheless, CGI-I remains the most widely used scale for defining responder criteria (Chang et al., 2021, Kim et al., 2022, Kim et al., 2015).

### 4.4 Model performance: Conventional vs. radiomics models

The reason why the CGI-I radiomics models outperformed conventional models only in children (although only during treatment) is intriguing. A key factor may be that children's brains are still developing, with regions like the basal ganglia among the last to mature (Bethlehem et al., 2022), leading to significant variability in volume during this developmental stage. Other features beyond volume may be more directly related to treatment response, indicating that the enhanced sensitivity of radiomics models likely stems from their ability to comprehensively analyze various characteristics of brain regions, such as shape, texture, and intensity; and this may be particularly valuable in small samples. Additionally, we can speculate that noise in images from children, e.g. introduced through motion, may have less impact on radiomics features compared to volumetric measures alone. Yet, the radiomics analysis was not robust to outlier removal, which could be due to the further reduction of an already small sample size. These speculations warrant further investigation in future studies.

While our most predictive model achieved a bAcc of 68 % and AUC-ROC of 0.73, other studies incorporating multiple modalities have reported higher predictive values. Kim et al. (2015) used a support vector machine with a comprehensive dataset including demographic, clinical questionnaires, other neuroimaging, and genetic information from 83 ADHD youths (9.5 ± 2.6 years, 65 boys), achieving an accuracy of 84.6 % and AUC-ROC of 0.84. Similarly, Chang et al. (2021) reached a bAcc of 87.4 % and AUC-ROC of 0.88 with a support vector machine analyzing baseline T1-weighted MRIs of 79 drug-naive individuals (aged 6–42 years). Several factors contribute to the disparity in our model performance below 0.80: differences in sample size and population diversity affecting generalizability, variations in MRI data quality such as motion artifacts and image resolution, and the use of a single imaging modality. Incorporating multimodal data could significantly enhance predictive power, as single-modality models may overlook critical genetic and clinical details vital for predicting treatment response.

### 4.5 Strengths and limitations

A potential strength of our study lies in pioneering the advantage of radiomics features over conventional volume features for predicting treatment response in ADHD participants. Several volume-based and voxel-based machine learning studies have been published in predicting ADHD treatment response (Chang et al., 2021, Kim et al., 2022, Kim et al., 2015, Parlatini et al., 2023). By focusing on first-order statistics and shape features extracted from ROIs, our analyses capture a broader range of morphological data than conventional ROI volume assessments, thereby potentially adding valuable neuroimaging insights to the explainability of the original data and prediction for MPH treatment efficacy.

Furthermore, our study underscores the importance of choosing the right strategy when constructing models with small sample sizes. In our subgroup analyses on children and adult groups during treatment using conventional and radiomics models, feature pre-selection ahead of XGBoost classification notably altered performance (see Supplement Table A4). In smaller samples, extensive feature selection may not necessarily enhance performance and might even reduce it. These observations are consistent with reports that using bootstrap data with the XGBoost classifier improves accuracy in small sample size scenarios, as opposed to feature selection and random data selection (Zhao and Duangsoithong, 2019). Notably, skipping the feature selection can also lead to model overfitting, which would require further validation using external data.

Due to the small sample sizes within the CGI-I good responder category, particularly among children, we were unable to conduct pediatric subgroup analyses. However, our model's performance was not significantly affected by the exclusion of three outliers with low-quality images when compared to the whole group. After excluding these outliers, bAcc and AUC-ROC values dropped slightly: for the conventional mode the bAcc was 59 % and AUC-ROC was 0.59; for the radiomics model, bAcc was 58 % and AUC-ROC was 0.57 (Wilcoxon signed-rank test, *P* = 0.55 for the conventional model and *P* = 0.33 for the radiomics model during treatment). To categorically correct for age group, we employed the ComBat method, a technique conceived for genomic data and later adapted for radiomics analyses, to harmonize the effects of age on imaging histological features (Horng et al., 2022, Mahon et al., 2020). Despite small sample sizes, we established radiomics models achieving moderate accuracy by separately analyzing the performance of the total cohort (n = 47), children (n = 23) and adults (n = 24). The balanced accuracy scores on radiomics features during treatment for these subgroups closely mirrored those from the total cohort, underlining the robustness of our findings across different age groups and confirming the efficacy of our approach in managing age-related biases.

While our study highlights the potential of both conventional and radiomics MRI features in predicting MPH response, the relatively low AUC suggests that the model is not yet suitable for clinical application. Future enhancements of our models could be achieved by incorporating additional clinical data and performing external validation in larger, independent cohorts to ensure generalizability. Also, our work selected ROIs based on a qualitative review of the literature. Future work may obtain a reliable quantitative summary through a *meta*-analysis.

## 5 Conclusion

Both conventional and radiomics models accurately predicted symptom improvement during treatment with MPH, while the prediction accuracy substantially declined for predicting treatment response one week after 16 weeks of MPH treatment. Radiomics features can offer enhanced structural information beyond conventional region-based volume and FA averages while maintaining adequate prediction accuracy, which we demonstrated here in the pediatric subgroup. Our results suggest that baseline basal ganglia structural radiomics information can provide valuable insights in predicting MPH medication response.

## 6 Data and code availability statement

The authors confirm that the data supporting the findings of this study are available within the article and its supplementary material. The documented code base is available on GitHub (https://github.com/Mancy-Chen/ePOD-MPH). Raw MRI data to support the findings of this study are available from the corresponding author, upon reasonable request.

## Declaration of Generative AI and AI-assisted technologies in the writing process

During the preparation of this work the authors used ChatGPT 4.0 in order to improve readability and language. After using ChatGPT 4.0, the authors reviewed and edited the content as needed and took full responsibility for the publication's content.

## CRediT authorship contribution statement

**Mingshi Chen:** Writing – original draft, Visualization, Validation, Investigation, Funding acquisition, Formal analysis. **Zarah van der Pal:** Writing – review & editing, Data curation. **Maarten G. Poirot:** Writing – review & editing, Methodology. **Anouk Schrantee:** Writing – review & editing, Data curation. **Marco Bottelier:** Writing – review & editing. **Sandra J.J. Kooij:** Writing – review & editing. **Henk A. Marquering:** Writing – review & editing, Supervision. **Liesbeth Reneman:** Funding acquisition, Writing – review & editing, Supervision, Resources, Project administration, Data curation, Conceptualization. **Matthan W.A. Caan:** Funding acquisition, Writing – review & editing, Supervision, Software.

## Funding

This research has received funding support from the China Scholarship Council (CSC), No.202206380049, and the ITEA (21016 DAIdnsortium.

## Declaration of competing interest

The authors declare the following financial interests/personal relationships which may be considered as potential competing interests: M. Chen is funded by the China Scholarship Council (CSC) from the Ministry of Education of P.R. China. M.W.A. Caan is a shareholder of Nico.lab International Ltd. H.A. Marquering is a co-founder and shareholder of Nico.lab International Ltd., TrianecT, and inSteps. The other authors declare that they have no known competing financial interests or personal relationships that could have appeared to influence the work reported in this paper.

## Data Availability

Data will be made available on request.
