# Supplementary material for: Prediction of methylphenidate treatment response for ADHD using conventional and radiomics T1 and DTI features: Secondary analysis of a randomized clinical trial
Source: Neuroimage Clin. 2024 Nov 21;45:103707. doi: 10.1016/j.nicl.2024.103707 (PMC11626811; doi:10.1016/j.nicl.2024.103707)
Supplement: Supplementary Data 1 [file mmc1.docx]

# **Appendix A**

**Supplemental methods**

**I.1 Study Design**

In the “effects of Psychotropic drugs On the Developing brain - methylphenidate” randomized controlled trial (ePOD-MPH RCT), we included 50 children (10-12 years of age) and 49 adult (23-30 years of age) male outpatients diagnosed with attention-deficit/hyperactivity disorder (ADHD; all subtypes) and in need of pharmacological therapy, as described elsewhere [(Schrantee et al., 2016)](https://paperpile.com/c/WPbuWw/xowVw). Participants were recruited through outpatient clinical programs at the Department of Child and Adolescent Psychiatry at Triversum (Alkmaar, the Netherlands), De Bascule Academic Center for Child and Adolescent Psychiatry (Amsterdam), and PsyQmental health facility (the Hague, the Netherlands).

Participants met strict criteria for ADHD diagnosis (all subtypes) according to the Diagnostic and Statistical Manual of Mental Disorders (DSM-IV, 4th edition), which was confirmed by a structured interview using the Diagnostic Interview Schedule for Children (NIMH-DISC-IV: authorized Dutch translation [(Ferdinand & van der Ende, 1998)](https://paperpile.com/c/WPbuWw/pXcC)) and the Diagnostic Interview for ADHD (DIVA 2.0) for adults [(Kooij & Francken, 2010)](https://paperpile.com/c/WPbuWw/bHoX). Exclusion criteria were: comorbid axis I psychiatric disorders needing medication at entry or general MRI contraindications, including electric implants or claustrophobia. Following the selection criteria, 47 ADHD participants (23 boys and 24 men) were included in our study.

**I.2 Image Quality Assurance**

The correction of distortions and motion in T1-weighted scans was measured in terms of the coefficient of joint variation (CJV) between gray matter and white matter. A lower value of CJV corresponds to less motion-induced artifacts [(Ganzetti et al., 2016)](https://paperpile.com/c/WPbuWw/n7tpG).

DTI image quality was determined based on an aggregate DTI motion score derived from logarithmic mean translation parameters of the coregistered diffusion-weighted images towards the non-diffusion-weighted image. To address distortions and motion in the images, we generated a corresponding motion score for each participant [(Mohammadi et al., 2010)](https://paperpile.com/c/WPbuWw/2GkTE). A lower value of the motion score indicates better image quality.

**I.3 Image Processing**

T1-weighted scans were segmented using FastSurfer with the Desikan-Killiany-Tourville atlas [(Henschel et al., 2020)](https://paperpile.com/c/WPbuWw/W3eGL). Based on prior research [(Nakao et al., 2011; Schrantee et al., 2020)](https://paperpile.com/c/WPbuWw/WC6yL+V1eTS), the identified regions of interest (ROIs) included the bilateral caudate, putamen, pallidum, and accumbens (basal ganglia). Based on our previous pre-clinical research [(van der Marel et al., 2015)](https://paperpile.com/c/WPbuWw/KAJhr) in which we found important (age-dependent) effects of MPH on the volume of the bilateral hippocampus in the rat, we added bilateral hippocampus, totaling 10 ROIs. Based on our previous (pre-clinical) research [(Bouziane et al., 2019; van der Marel et al., 2014)](https://paperpile.com/c/WPbuWw/i9UFn+2s9CF) in which we observed the effects of MPH on developing white matter, we also included FA values of the entire brain white matter, bilateral Anterior Thalamic Radiation (ATR), and the splenium of the corpus callosum, amounting to 4 ROIs for the DTI analysis, for which the Johns Hopkins University atlas for atlas-based segmentation was used [(Mori et al., 2005; Wakana et al., 2007)](https://paperpile.com/c/WPbuWw/lU24t+x3vLw).

In light of findings from our prior research [(Poirot et al., 2022)](https://paperpile.com/c/WPbuWw/9ufLi), which demonstrated that shape-based and first-order statistics generally exhibit better reproducibility than higher-order features, we limited our study to these two classes of radiomics features. To explore the efficacy of predictive features at varying scales, we grouped the analyses into two categories that share the same ROI, each defined by their respective level of scientific evidence [(Poirot et al., 2024)](https://paperpile.com/c/WPbuWw/gpTp8). Radiomics features were extracted using Pyradiomics 3.1.0, capturing first-order statistics and shape-based features from T1 [(van Griethuysen et al., 2017)](https://paperpile.com/c/WPbuWw/SAUn5). For DTI, we only included first-order statistics, as shape-based features were not applicable to skeletonized FA maps in the TBSS analysis.

**1.4 Statistical Analyses**

In the Bayesian Estimation Supersedes the t-test (BEST) with a 95% Highest Density Interval (HDI) as a confidence interval, statistical significance was indicated if the effect size's 95% HDI -did not include 0.

To gain insights into each model's feature contribution, SHapley Additive exPlanations (SHAP) values were generated from the training set [(Lundberg et al., 2020)](https://paperpile.com/c/WPbuWw/34GNA). The ten most relevant features over all outer cross-validation loops were selected based on the highest absolute Shapley value computed on the training data. Given the small number of discordant pairs, exact binomial tests (one-tail) were performed to evaluate models’ performances between conventional models vs. chance, radiomics models vs. chance, and conventional vs. radiomics models [(Sundjaja et al., 2023)](https://paperpile.com/c/WPbuWw/Y7jHN). We statistically tested differences between good and poor responders in various datasets: the total cohort and subgroups of children and adults respectively, to investigate whether our prediction models’ performance differs between children and adults.

**Table A.1 Features for conventional and radiomics analyses.**

| Analysis model | T1 | | DTI | |
| --- | --- | --- | --- | --- |
|  | Category | Item | Category | Item |
| Conventional model | Shape-based ROI | Volume of ROI | First-order statistics of FA | Mean FA |
| Radiomics model | First-order statistics of ROI | 1. 10 Percentile 2. 90 Percentile 3. Energy 4. Entropy 5. Interquartile Range 6. Kurtosis 7. Maximum 8. Mean Absolute Deviation 9. Mean 10. Median 11. Minimum 12. Range 13. Robust Mean Absolute Deviation 14. Root Mean Squared 15. Skewness 16. Total Energy 17. Uniformity 18. Variance | First-order statistics of FA | 1. 10 Percentile 2. 90 Percentile 3. Energy 4. Interquartile Range 5. Kurtosis 6. Maximum 7. Mean Absolute Deviation 8. Mean 9. Median 10. Range 11. Robust Mean Absolute Deviation 12. Root Mean Squared 13. Skewness 14. Total Energy 15. Variance |
|  | Shape-based ROI | 1. Elongation 2. Flatness 3. Least Axis Length 4. Major Axis Length 5. Maximum 2D Diameter Column 6. Maximum 2D Diameter Row 7. Maximum 2D Diameter Slice 8. Maximum 3D Diameter 9. Mesh Volume 10. Minor Axis Length 11. Sphericity 12. Surface Area 13. Surface Volume Ratio 14. Voxel Volume |  |  |

Note: Zero or invariant features were removed.

Conventional analyses total features: 10 (T1) + 4 (DTI) = 14

Radiomics analyses total features: 10 × 32 (T1) + 4 × 15 (DTI) = 380

**Table A.2 Response rate of CGI-I.**

| Criteria | Endpoint | Response rate  (Good treatment responders/Total participants) | | |
| --- | --- | --- | --- | --- |
|  |  | Children | Adults | Total |
| CGI-I | During treatment | 7/23  (30.4%) | 10/24  (41.7%) | 17/47  (36.2%) |
|  | Post-treatment | 3/23  (13.0%) | 15/24  (62.5%) | 18/47  (38.3%) |

CGI-I: Clinical Global Impressions - Improvement, good treatment responders are defined as having scores of 1 or 2.

**Table A.3 The proportion of low symptom severity rate of CGI-S.**

| Criteria | Endpoint | Proportion of low symptom severity rate  (Low symptom severity participants/Total participants) | | |
| --- | --- | --- | --- | --- |
|  |  | Children | Adults | Total |
| CGI-S | Baseline | 0/23  (0.0%) | 4/24  (16.7%) | 4/47  (8.5%) |
|  | During treatment | 8/23  (34.8%) | 20/24  (83.3%) | 28/47  (59.6%) |
|  | Post-treatment | 6/23  (26.1%) | 17/24  (70.8%) | 23/47  (48.9%) |

CGI-S: Clinical Global Impressions - Severity, low symptom severity participants are defined as having scores of 1, 2 or 3.

**Table A.4 Sensitivity analyses of conventional and radiomics model performance with CGI-I as outcome evaluation.**

|  |  | Conventional model | | | | | | Conventional model vs. chance | Radiomics model | | | | | | Radiomics model vs. chance | Conventional vs. radiomics model |
| --- | --- | --- | --- | --- | --- | --- | --- | --- | --- | --- | --- | --- | --- | --- | --- | --- |
| Endpoint | Group | bAcc ^a^  (%) | Precision | Recall | F1 Score | AUC-  ROC ^b^ | AUC–PRC ^c^ | *P* value ^d^ | bAcc ^a^  (%) | Precision | Recall | F1 Score | AUC-  ROC ^b^ | AUC-PRC ^c^ | *P* value ^d^ | *P* value ^d^ |
| During treatment | 47 children + adults ^e^ | 54 | 0.41 | 0.41 | 0.41 | 0.63 | 0.49 | 0.38 | 65 | 0.56 | 0.53 | 0.55 | 0.67 | 0.57 | 0.02 | 0.27 |
|  | 23 children - FS ^f^ | 23 | 0.08 | 0.14 | 0.11 | 0.25 | 0.23 | 0.03 | 50 | 0.31 | 0.57 | 0.40 | 0.46 | 0.34 | 1.00 | 0.18 |
|  | 24 adults - FS ^f^ | 46 | 0.38 | 0.50 | 0.43 | 0.46 | 0.47 | 0.84 | 70 | 0.56 | 0.90 | 0.69 | 0.89 | 0.89 | 0.15 | 0.33 |
|  | 44 children + adults - FS ^f,h^ | 59 | 0.48 | 0.59 | 0.53 | 0.59 | 0.51 | 0.29 | 58 | 0.47 | 0.53 | 0.50 | 0.57 | 0.45 | 0.29 | 1.00 |
|  | 20 children ^g,h^ | 26 | 0.11 | 0.14 | 0.12 | 0.31 | 0.30 | 0.12 | 34 | 0.14 | 0.14 | 0.14 | 0.36 | 0.41 | 0.50 | 0.73 |
|  | 20 children - FS ^f,h^ | 58 | 0.40 | 0.86 | 0.55 | 0.64 | 0.52 | 1.00 | 30 | 0.18 | 0.29 | 0.22 | 0.38 | 0.31 | 0.12 | 0.34 |
| Post-treatment | 47 children + adults ^e^ | 47 | 0.35 | 0.39 | 0.37 | 0.40 | 0.35 | 1.00 | 36 | 0.29 | 0.56 | 0.38 | 0.45 | 0.43 | 0.02 | 0.13 |
|  | 23 children - FS ^f,i^ | - | - | - | - | - | - | - | - | - | - | - | - | - | - | - |
|  | 24 adults - FS ^f^ | 43 | 0.57 | 0.53 | 0.55 | 0.37 | 0.60 | 0.84 | 53 | 0.65 | 0.73 | 0.69 | 0.58 | 0.70 | 0.54 | 0.58 |
|  | 44 children + adults - FS ^f,h^ | 38 | 0.27 | 0.35 | 0.31 | 0.47 | 0.45 | 0.17 | 42 | 0.30 | 0.35 | 0.32 | 0.38 | 0.38 | 0.45 | 0.83 |
|  | 20 children ^g,h,i^ | - | - | - | - | - | - | - | - | - | - | - | - | - | - | - |
|  | 20 children - FS ^f,h,i^ | - | - | - | - | - | - | - | - | - | - | - | - | - | - | - |

^a^: bAcc: Balanced accuracy

^b^: AUC-ROC: Area under the Receiver Operating Characteristic Curve.

^c^: AUC-PRC: Area under the Precision-Recall Curve.

^d^: *P* values were obtained by exact binomial tests.

^e^: No feature selection in total cohort analyses with 47 participants.

^f^: FS: feature selection. 5 features were selected in 23 and 24 participants’ subgroup analyses, 7 were selected in 44 participants, and 4 in 20 participants. No ComBat harmonization was applied.

^g^: No ComBat harmonization and no feature selection in subgroup analyses with 20 children.

^h^ : 3 outliers were excluded for sensitivity analysis.

^i^: Post-treatment CGI-I with children subgroup analyses had limited good responders (3/23, 2/20), and it failed to classify.

**Table A.5 Conventional and radiomics model performance with CGI-S as outcome evaluation.**

|  |  |  | Conventional model | | | | | | Conventio-  nal model vs. chance |  | Radiomics model | | | | | | Radiomics model vs. chance |  | Conventional vs. radiomics model |
| --- | --- | --- | --- | --- | --- | --- | --- | --- | --- | --- | --- | --- | --- | --- | --- | --- | --- | --- | --- |
| Endpoint | Group |  | bAcc ^a^  (%) | Precision | Recall | F1 Score | AUC-ROC ^b^ | AUC-PRC ^c^ | *P* value ^d^ |  | bAcc ^a^  (%) | Precision | Recall | F1 Score | AUC-  ROC ^b^ | AUC-  PRC ^c^ | *P* value ^d^ |  | *P* value ^d^ |
| During treatment | 47 children + adults (FS) ^e^ |  | 37 | 0.58 | 0.68 | 0.63 | 0.39 | 0.61 | 0.77 |  | 49 | 0.65 | 0.55 | 0.60 | 0.47 | 0.66 | 1.00 |  | 0.83 |
|  | 23 children ^f^ |  | 29 | 0.17 | 0.25 | 0.20 | 0.33 | 0.29 | 0.09 |  | 45 | 0.30 | 0.38 | 0.33 | 0.48 | 0.35 | 1.00 |  | 0.39 |
|  | 24 adults ^f^ |  | 50 | 0.83 | 1.00 | 0.91 | 0.50 | 0.83 | 0.002 |  | 50 | 0.83 | 1.00 | 0.91 | 0.50 | 0.83 | 0.002 |  | 1.00 |
| Post-  treatment | 47 children + adults (FS) ^e^ |  | 66 | 0.60 | 0.71 | 0.65 | 0.63 | 0.57 | 0.04 |  | 70 | 0.67 | 0.67 | 0.67 | 0.73 | 0.67 | 0.008 |  | 0.77 |
|  | 23 children ^f^ |  | 57 | 0.31 | 0.67 | 0.42 | 0.49 | 0.28 | 1.00 |  | 68 | 0.38 | 0.83 | 0.53 | 0.54 | 0.38 | 0.40 |  | 0.69 |
|  | 24 adults ^f^ |  | 84 | 0.93 | 0.82 | 0.87 | 0.77 | 0.83 | 0.002 |  | 50 | 0.71 | 0.71 | 0.71 | 0.61 | 0.85 | 0.54 |  | 0.07 |

^a^: bAcc: Balanced accuracy

^b^: AUC-ROC: Area under the Receiver Operating Characteristic Curve.

^c^: AUC-PRC: Area under the Precision-Recall Curve.

^d^: *P* values were obtained by exact binomial tests

^e^: FS: feature selection. 7 features were selected in 47 participants (children+adults).

^f^: No ComBat harmonization and no feature selection in subgroup analyses.

**131** Assessed for eligibility

(75 children and 56 adults)

**32** Excluded (25 children and 7 adults)

**24** Did not meet inclusion criteria

(19 children and 5 adults)

**8** Declined to participate

(6 children and 2 adults)

**50** Received methylphenidate

(25 children and 25 adults)

**49** Received placebo

(25 children and 24 adults)

**47** Entered in the secondary analysis

(**23** children and **24** adults)

**2** Children and

**1** Adults excluded

(Lack of TBSS data)

**Figure A.1 CONSORT diagram of the inclusion process**


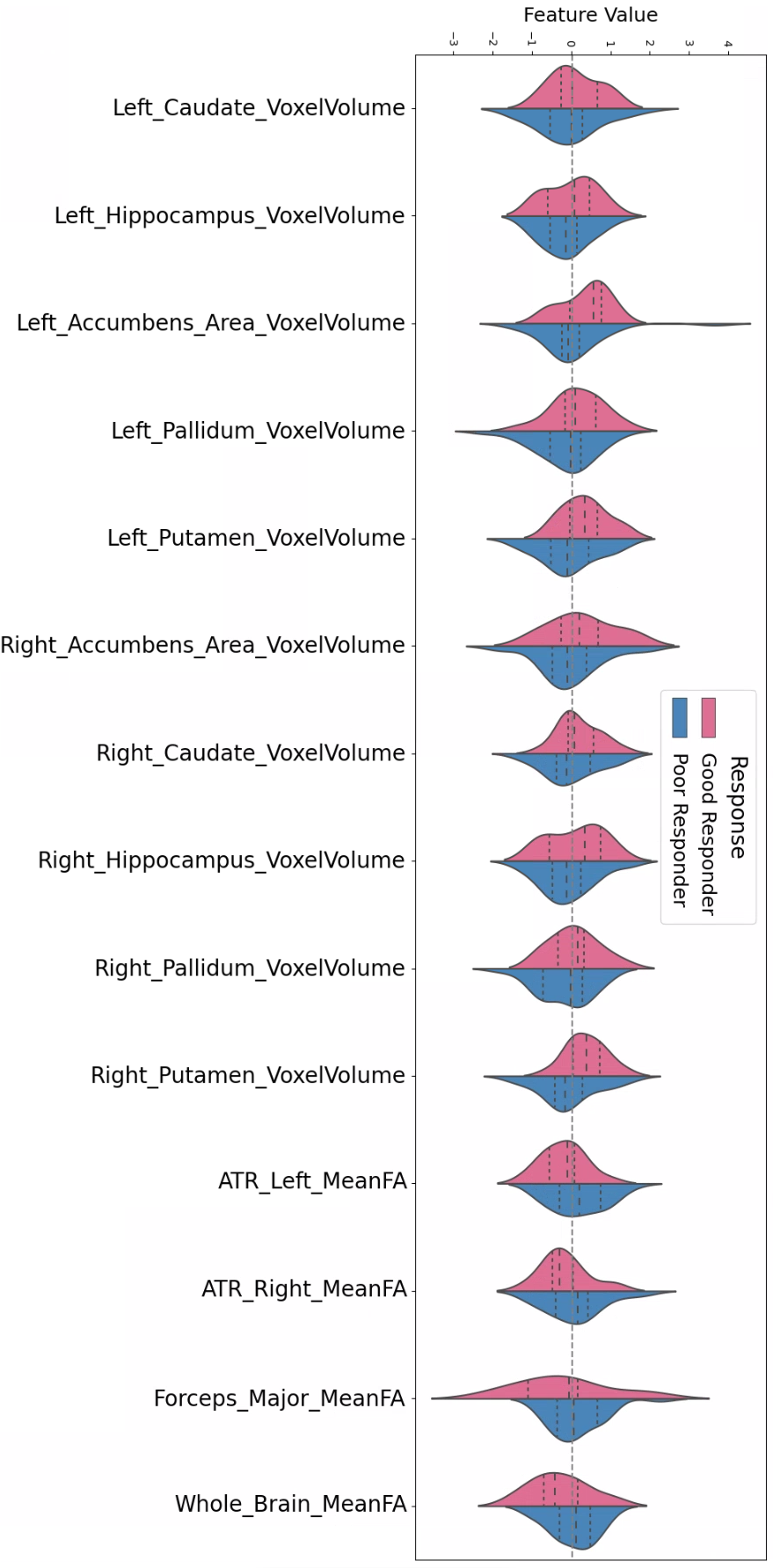


**Figure A.2 Violin plot of all regional volumes or mean FA values MRI features with during treatment CGI-I as treatment outcome.** Feature values underwent scaling and were harmonized using the NeuroComBat method. Dashed line: upper (75th percentile) quartiles, median (50th percentile), and lower (25th percentile) quartiles.


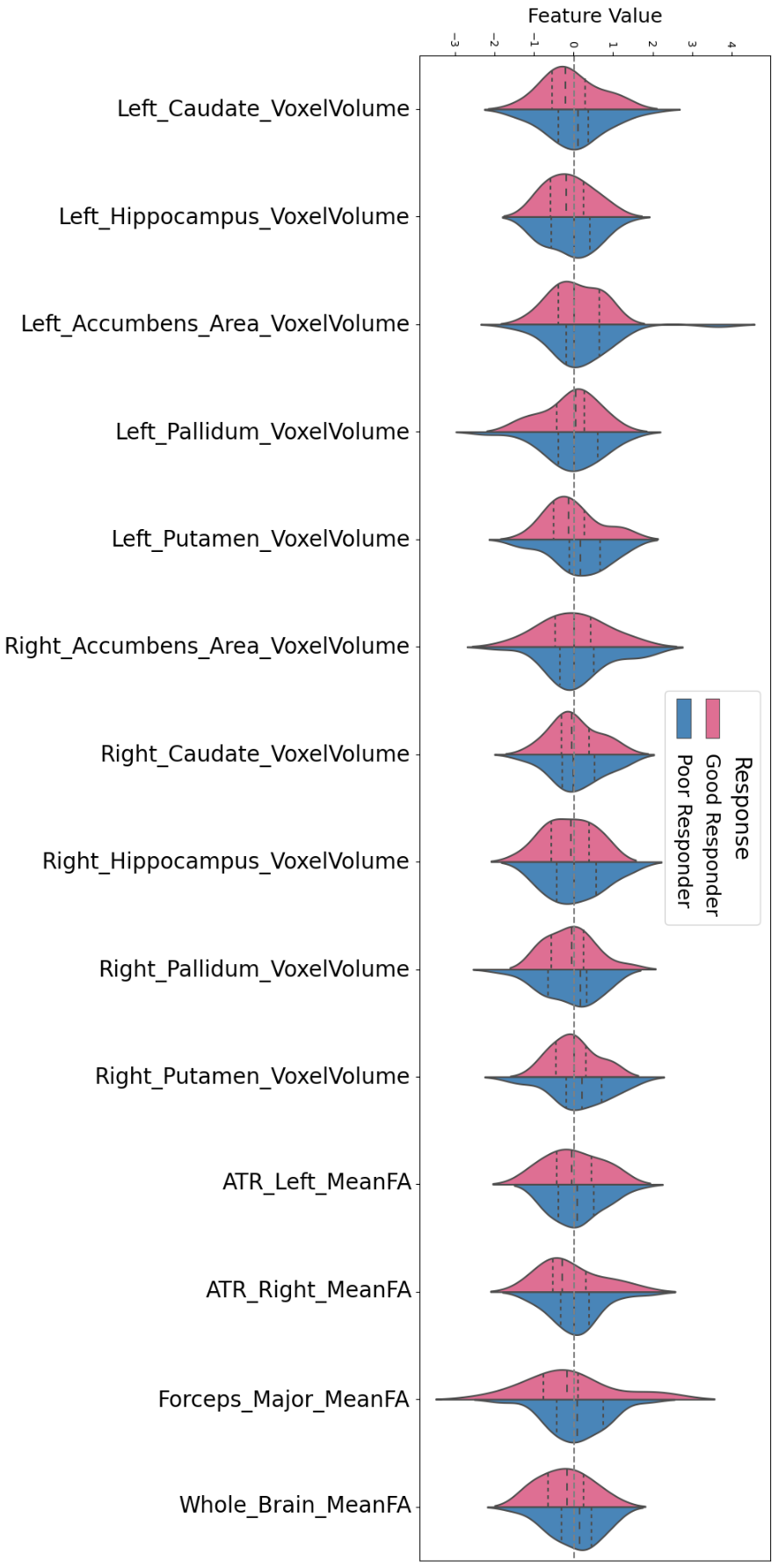


**Figure A.3 Violin plot of all regional volumes or mean FA values MRI features with post-treatment CGI-I as treatment outcome.** Feature values underwent scaling and were harmonized using the NeuroComBat method. Dashed line: upper (75th percentile) quartiles, median (50th percentile), and lower (25th percentile) quartiles.


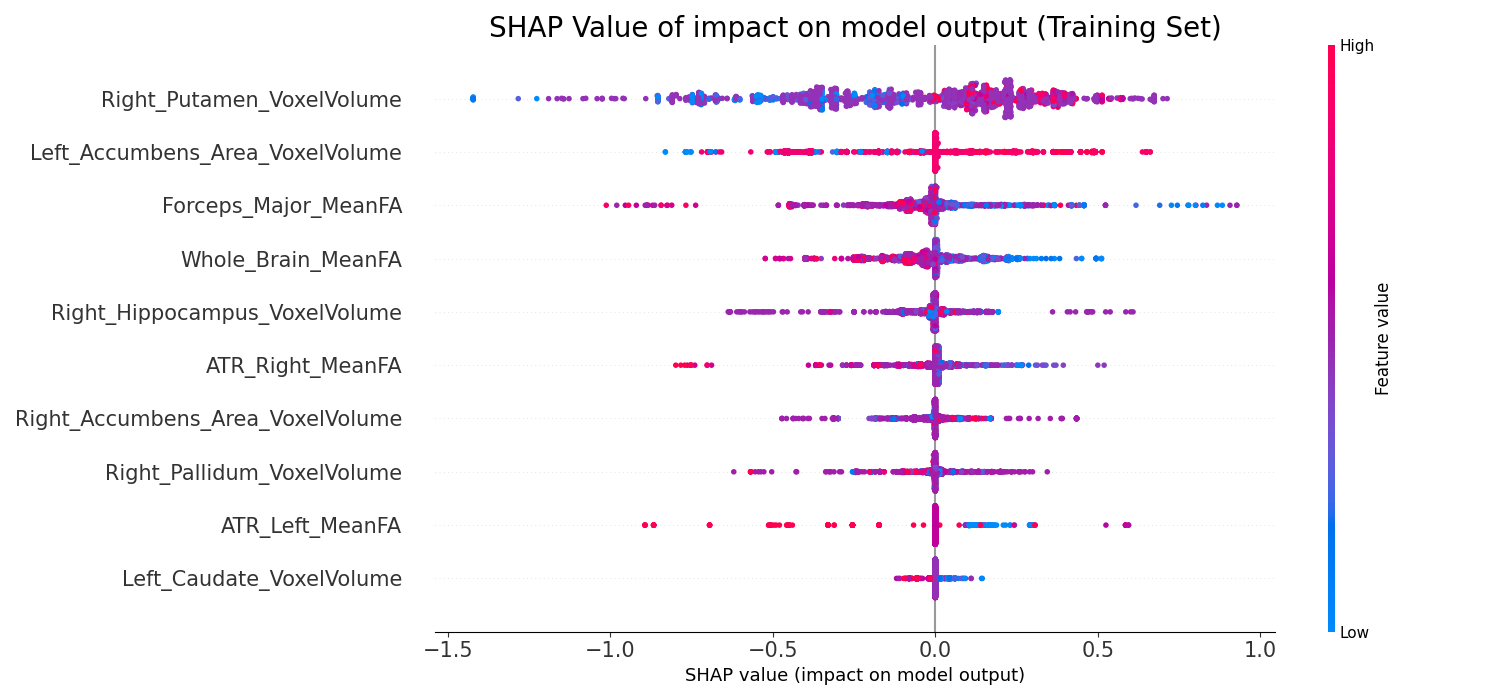


**Figure A.4 Top 10 SHapley Additive exPlanations (SHAP) values of conventional models in training set for the 47 total cohort during treatment with CGI-I evaluation.**


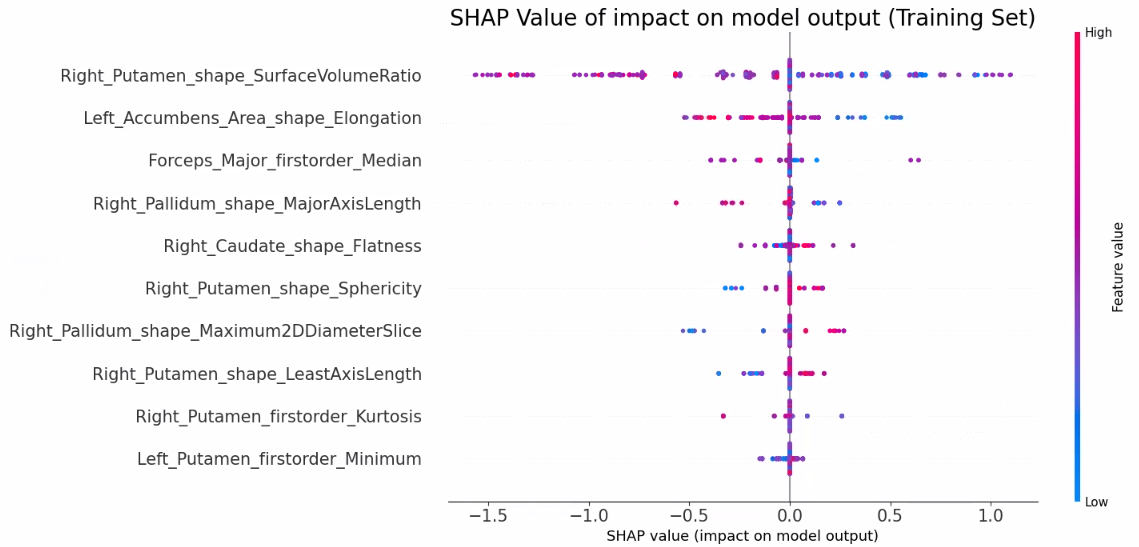


**Figure A.5 Top 10 SHapley Additive exPlanations (SHAP) values of radiomics models in training set for 23 children during treatment with CGI-I evaluation.**

**Figure A.6 Top 10 SHapley Additive exPlanations (SHAP) values of radiomics models in training set for 24 adults during treatment with CGI-I evaluation.**
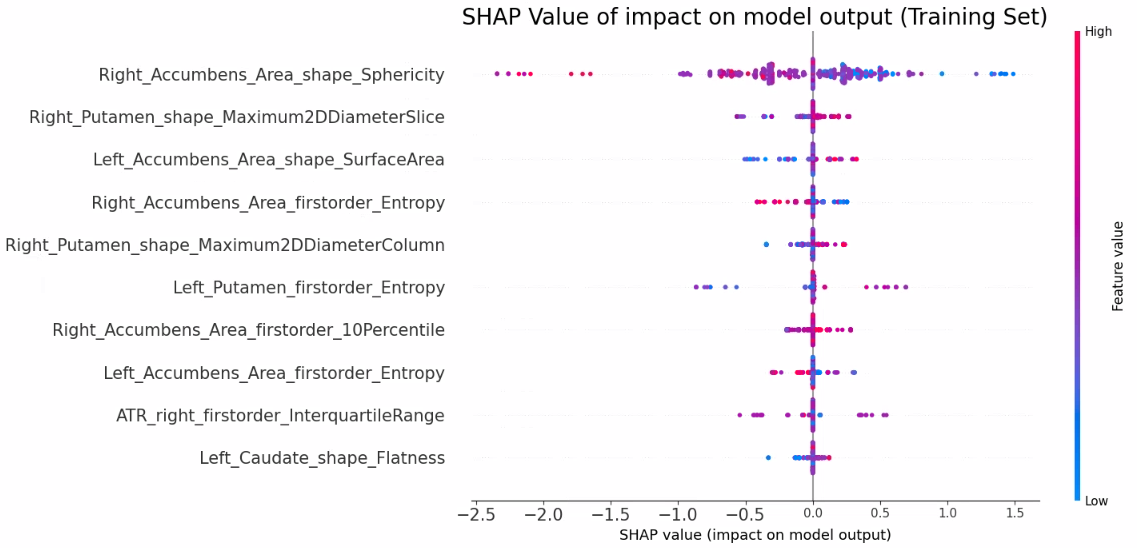


**
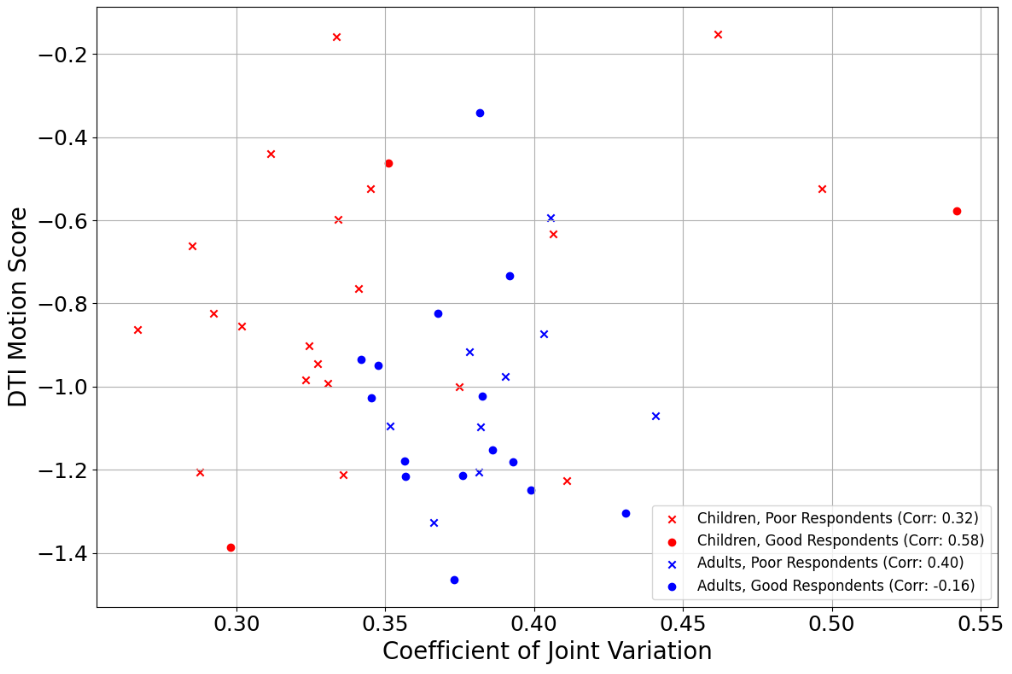
**

**Figure A.7 Scatter plot with correlation analysis between Coefficient of Joint Variation and motion score based on during treatment CGI-I evaluation.** Three outliers from the children's subgroup can be observed in the upper right corner.


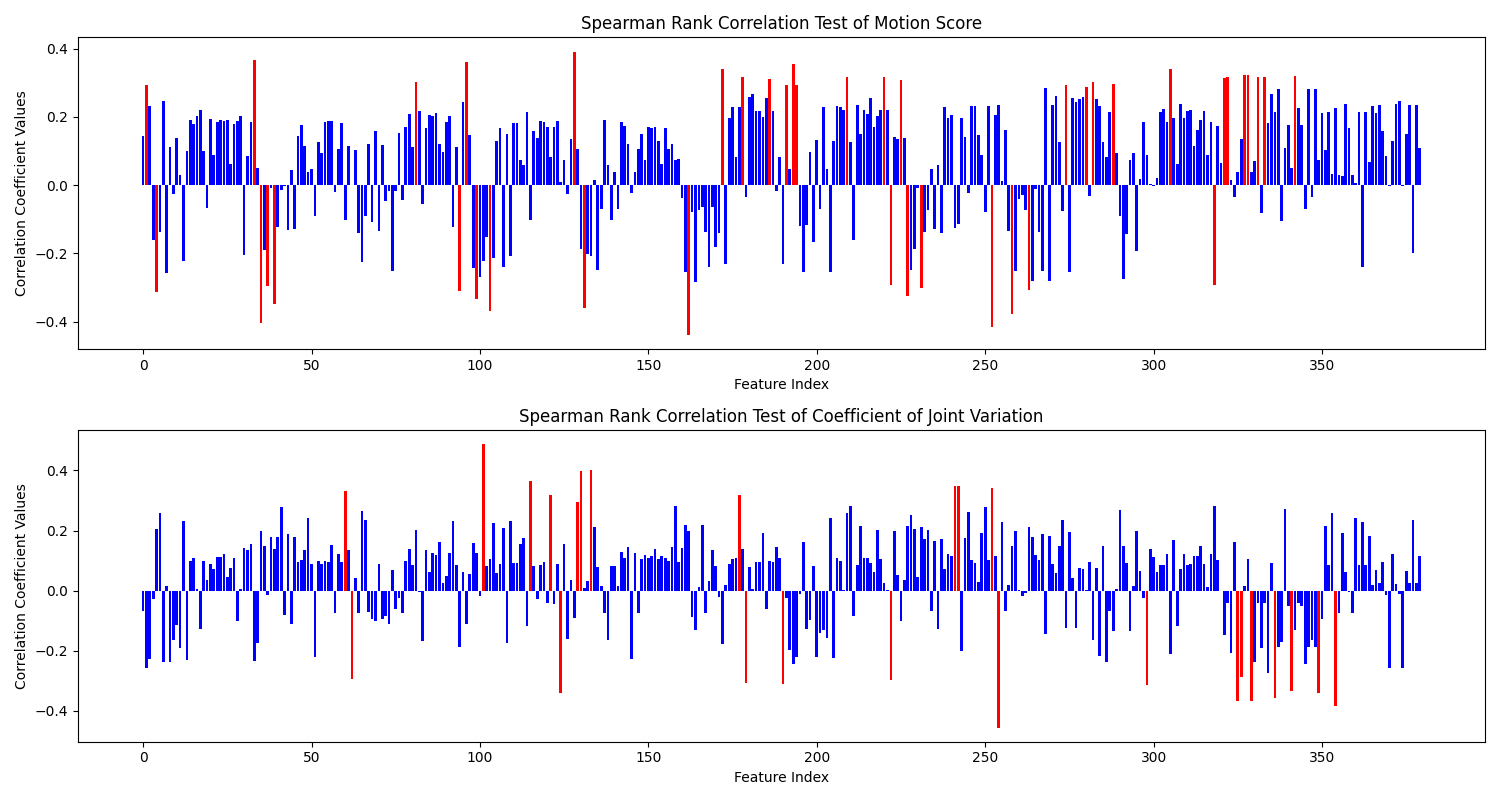

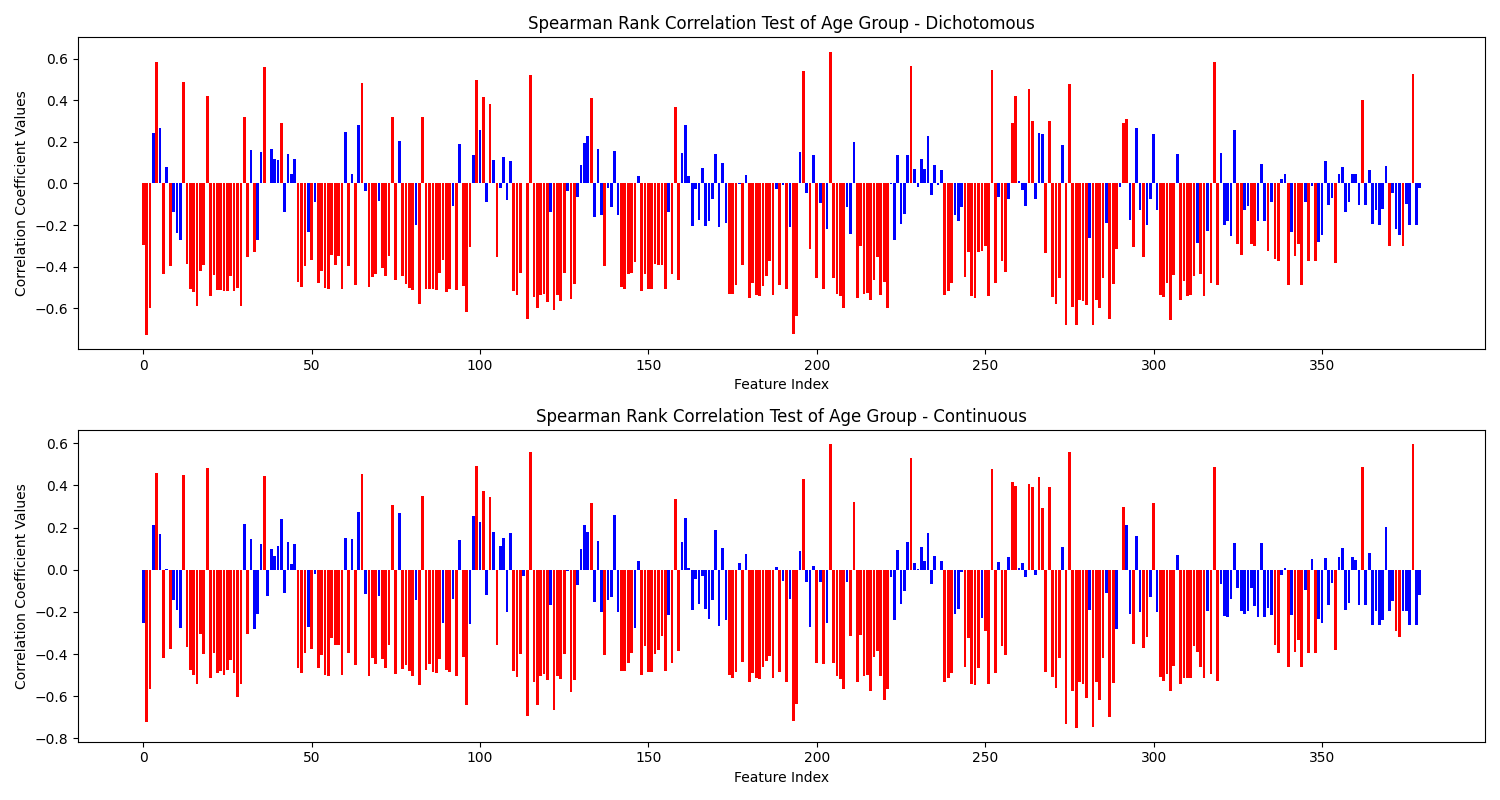


**Figure A.8 Spearman Rank Correlation Test with no harmonization of 380 features in the total cohort (n = 47).** The graphs show the 380 features’ correlation with age group (children/adults), DTI motion score, and coefficient of joint variation (CJV), respectively (Red lines: *P* < 0.05, blue lines: *P* ≥ 0.05).


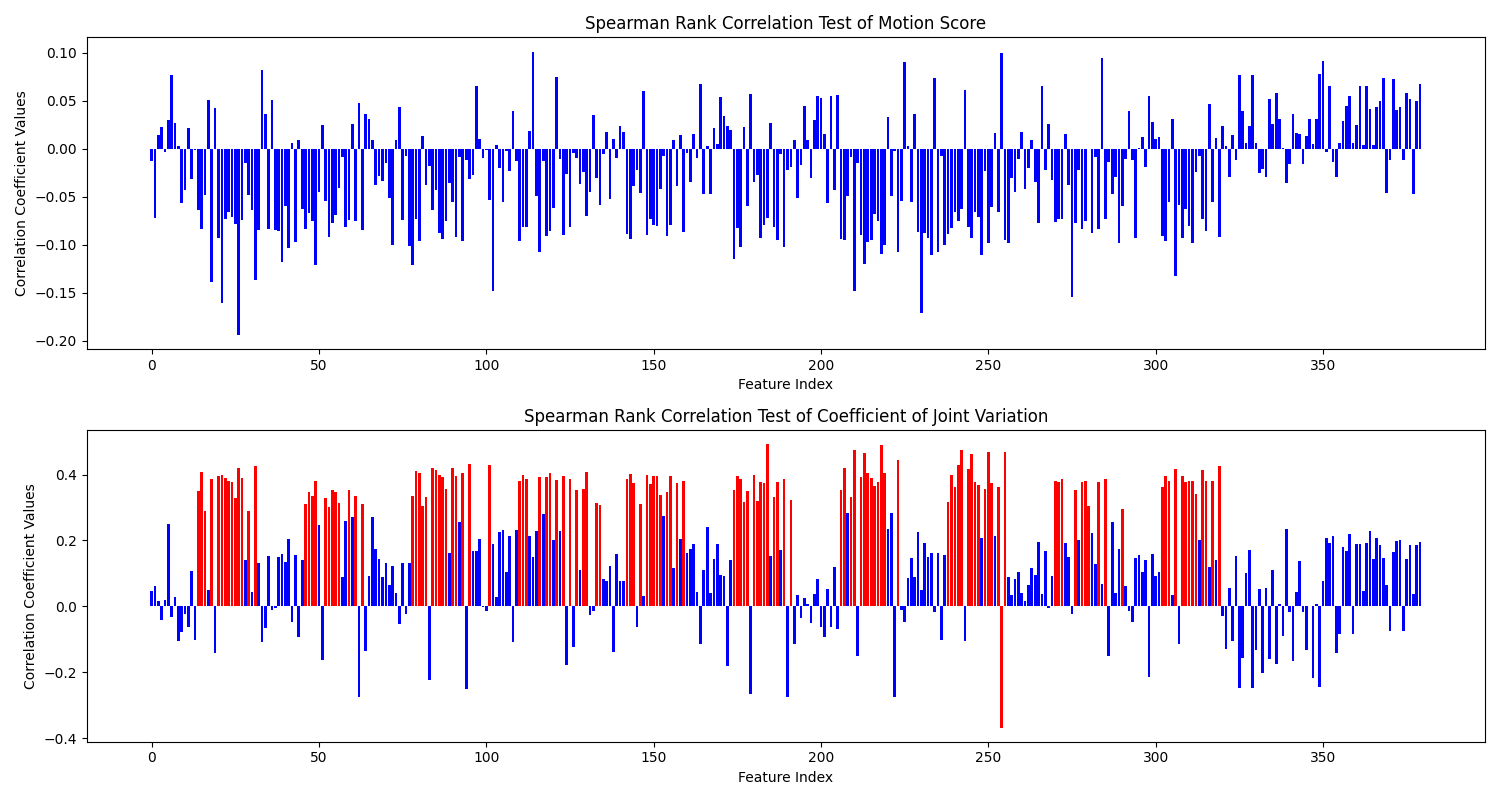

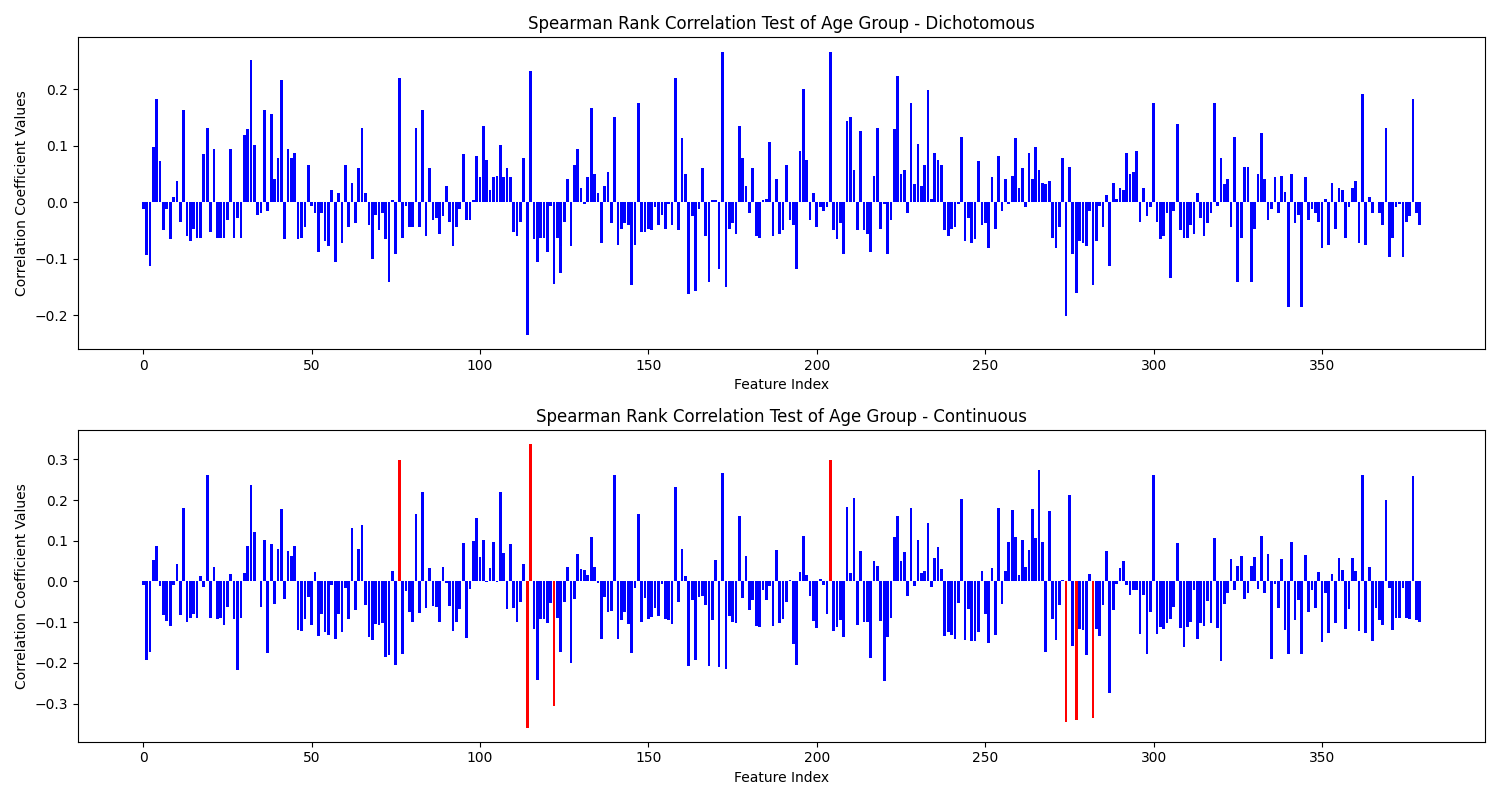


**Figure A.9 Spearman Rank Correlation Test with ComBat and GAMs harmonization of 380 features in the total cohort (n = 47).** The graphs show the 380 features’ correlation with age group (children/adults), DTI motion score, and coefficient of joint variation (CJV), respectively (Red lines: *P* < 0.05, blue lines: *P* ≥ 0.05).


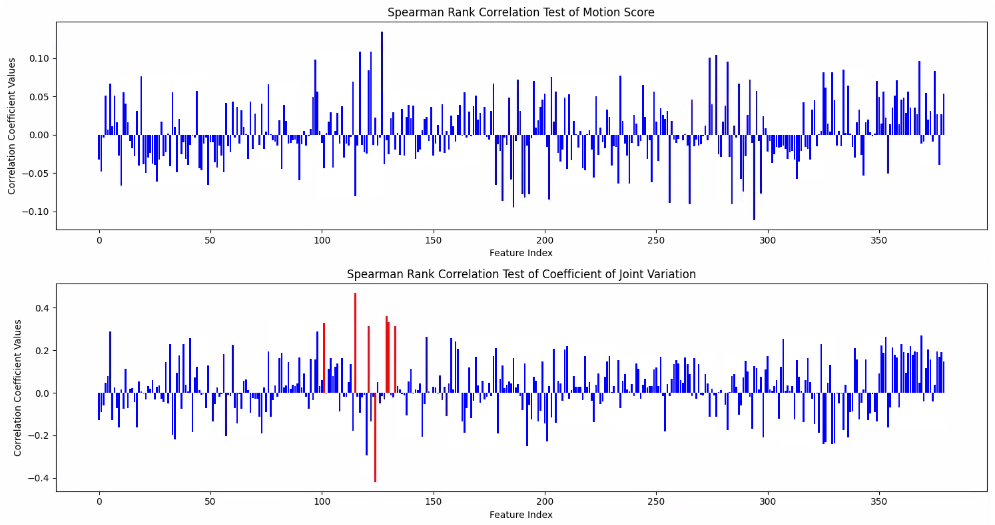

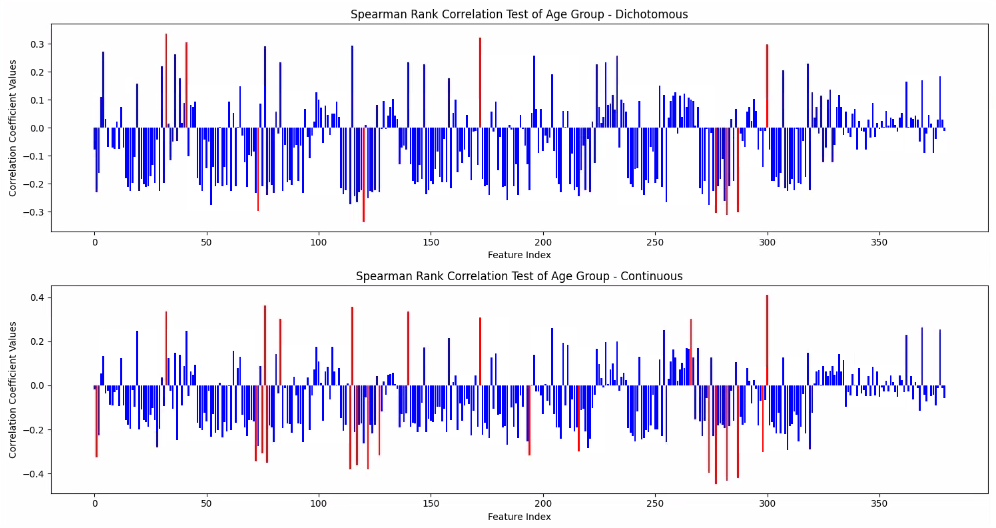


**Figure A.10 Spearman Rank Correlation Test with ComBat and GAMs harmonization of 380 features in the total cohort excluding the three outliers (n = 44).** The graphs show the 380 features’ correlation with age group (children/adults), DTI motion score, and coefficient of joint variation (CJV), respectively (Red lines: *P* < 0.05, blue lines: *P* ≥ 0.05).

**SUPPLEMENTAL REFERENCES**

[Bouziane, C., Filatova, O. G., Schrantee, A., Caan, M. W. A., Vos, F. M., & Reneman, L. (2019). White Matter by Diffusion MRI Following Methylphenidate Treatment: A Randomized Control Trial in Males with Attention-Deficit/Hyperactivity Disorder. *Radiology*, *293*(1), 186–192.](http://paperpile.com/b/WPbuWw/2s9CF)

[Ferdinand, R. F., & van der Ende, J. (1998). *DISC-IV: diagnostic interview schedule for children : Nederlandse vertaling NIMH-DISC-IV*.](http://paperpile.com/b/WPbuWw/pXcC)

[Ganzetti, M., Wenderoth, N., & Mantini, D. (2016). Intensity Inhomogeneity Correction of Structural MR Images: A Data-Driven Approach to Define Input Algorithm Parameters. *Frontiers in Neuroinformatics*, *10*, 10.](http://paperpile.com/b/WPbuWw/n7tpG)

[Henschel, L., Conjeti, S., Estrada, S., Diers, K., Fischl, B., & Reuter, M. (2020). FastSurfer - A fast and accurate deep learning based neuroimaging pipeline. *NeuroImage*, *219*, 117012.](http://paperpile.com/b/WPbuWw/W3eGL)

[Kooij, J. J. S., & Francken, M. H. (2010). *Diagnostic Interview for ADHD in adults (DIVA) 2.0*. Diagnostic Interview for ADHD in Adults (DIVA) 2.0.](http://paperpile.com/b/WPbuWw/bHoX) <https://www.advancedassessments.co.uk/resources/ADHD-Screening-Test-Adult.pdf>

[Lundberg, S. M., Erion, G., Chen, H., DeGrave, A., Prutkin, J. M., Nair, B., Katz, R., Himmelfarb, J., Bansal, N., & Lee, S.-I. (2020). From Local Explanations to Global Understanding with Explainable AI for Trees. *Nature Machine Intelligence*, *2*(1), 56–67.](http://paperpile.com/b/WPbuWw/34GNA)

[Mohammadi, S., Möller, H. E., Kugel, H., Müller, D. K., & Deppe, M. (2010). Correcting eddy current and motion effects by affine whole-brain registrations: evaluation of three-dimensional distortions and comparison with slicewise correction. *Magnetic Resonance in Medicine: Official Journal of the Society of Magnetic Resonance in Medicine / Society of Magnetic Resonance in Medicine*, *64*(4), 1047–1056.](http://paperpile.com/b/WPbuWw/2GkTE)

[Mori, S., Wakana, S., van Zijl, P. C. M., & Nagae-Poetscher, L. M. (2005). *MRI Atlas of Human White Matter*. Elsevier.](http://paperpile.com/b/WPbuWw/lU24t)

[Nakao, T., Radua, J., Rubia, K., & Mataix-Cols, D. (2011). Gray matter volume abnormalities in ADHD: voxel-based meta-analysis exploring the effects of age and stimulant medication. *The American Journal of Psychiatry*, *168*(11), 1154–1163.](http://paperpile.com/b/WPbuWw/V1eTS)

[Poirot, M. G., Caan, M. W. A., Ruhe, H. G., Bjørnerud, A., Groote, I., Reneman, L., & Marquering, H. A. (2022). Robustness of radiomics to variations in segmentation methods in multimodal brain MRI. *Scientific Reports*, *12*(1), 1–10.](http://paperpile.com/b/WPbuWw/9ufLi)

[Poirot, M. G., Ruhe, H. G., Mutsaerts, H.-J. M. M., Maximov, I. I., Groote, I. R., Bjørnerud, A., Marquering, H. A., Reneman, L., & Caan, M. W. A. (2024). Treatment Response Prediction in Major Depressive Disorder Using Multimodal MRI and Clinical Data: Secondary Analysis of a Randomized Clinical Trial. *The American Journal of Psychiatry*, *181*(3), 223–233.](http://paperpile.com/b/WPbuWw/gpTp8)

[Schrantee, A., Ruhé, H. G., & Reneman, L. (2020). Psychoradiological Biomarkers for Psychopharmaceutical Effects. *Neuroimaging Clinics of North America*, *30*(1), 53–63.](http://paperpile.com/b/WPbuWw/WC6yL)

[Schrantee, A., Tamminga, H. G. H., Bouziane, C., Bottelier, M. A., Bron, E. E., Mutsaerts, H.-J. M. M., Zwinderman, A. H., Groote, I. R., Rombouts, S. A. R. B., Lindauer, R. J. L., Klein, S., Niessen, W. J., Opmeer, B. C., Boer, F., Lucassen, P. J., Andersen, S. L., Geurts, H. M., & Reneman, L. (2016). Age-Dependent Effects of Methylphenidate on the Human Dopaminergic System in Young vs Adult Patients With Attention-Deficit/Hyperactivity Disorder: A Randomized Clinical Trial. *JAMA Psychiatry* , *73*(9), 955–962.](http://paperpile.com/b/WPbuWw/xowVw)

[Sundjaja, J. H., Shrestha, R., & Krishan, K. (2023). McNemar And Mann-Whitney U Tests. In *StatPearls [Internet]*. StatPearls Publishing.](http://paperpile.com/b/WPbuWw/Y7jHN)

[van der Marel, K., Bouet, V., Meerhoff, G. F., Freret, T., Boulouard, M., Dauphin, F., Klomp, A., Lucassen, P. J., Homberg, J. R., Dijkhuizen, R. M., & Reneman, L. (2015). Effects of long-term methylphenidate treatment in adolescent and adult rats on hippocampal shape, functional connectivity and adult neurogenesis. *Neuroscience*, *309*, 243–258.](http://paperpile.com/b/WPbuWw/KAJhr)

[van der Marel, K., Klomp, A., Meerhoff, G. F., Schipper, P., Lucassen, P. J., Homberg, J. R., Dijkhuizen, R. M., & Reneman, L. (2014). Long-term oral methylphenidate treatment in adolescent and adult rats: differential effects on brain morphology and function. *Neuropsychopharmacology: Official Publication of the American College of Neuropsychopharmacology*, *39*(2), 263–273.](http://paperpile.com/b/WPbuWw/i9UFn)

[van Griethuysen, J. J. M., Fedorov, A., Parmar, C., Hosny, A., Aucoin, N., Narayan, V., Beets-Tan, R. G. H., Fillion-Robin, J.-C., Pieper, S., & Aerts, H. J. W. L. (2017). Computational Radiomics System to Decode the Radiographic Phenotype. *Cancer Research*, *77*(21), e104–e107.](http://paperpile.com/b/WPbuWw/SAUn5)

[Wakana, S., Caprihan, A., Panzenboeck, M. M., Fallon, J. H., Perry, M., Gollub, R. L., Hua, K., Zhang, J., Jiang, H., Dubey, P., Blitz, A., van Zijl, P., & Mori, S. (2007). Reproducibility of quantitative tractography methods applied to cerebral white matter. *NeuroImage*, *36*(3), 630–644.](http://paperpile.com/b/WPbuWw/x3vLw)
